# ER+, HER2− advanced breast cancer treated with taselisib and fulvestrant: genomic landscape and associated clinical outcomes

**DOI:** 10.1002/1878-0261.13416

**Published:** 2023-03-25

**Authors:** Jessica W. Chen, William Jacot, Javier Cortés, Ian E. Krop, Susan Dent, Nadia Harbeck, Michelino De Laurentiis, Véronique Diéras, Young‐Hyuck Im, Thomas J. Stout, Frauke Schimmoller, Heidi M. Savage, Katherine E. Hutchinson, Timothy R. Wilson

**Affiliations:** ^1^ Oncology Biomarker Development Genentech, Inc. South San Francisco CA USA; ^2^ Institut du Cancer de Montpellier (ICM) Val d'Aurelle Montpellier University, INSERM U1194 France; ^3^ International Breast Cancer Center (IBCC), Pangaea Oncology Quironsalud Group Madrid Spain; ^4^ International Breast Cancer Center (IBCC), Pangaea Oncology Quironsalud Group Barcelona Spain; ^5^ Faculty of Biomedical and Health Sciences, Department of Medicine Universidad Europea de Madrid Madrid Spain; ^6^ Yale Cancer Center New Haven CT USA; ^7^ Duke Cancer Institute Duke University Durham NC USA; ^8^ Breast Center, Department Gynecology and Obstetrics and Comprehensive Cancer Center (CCC) Munich Ludwig‐Maximilians‐University (LMU) Hospital Munich Germany; ^9^ Istituto Nazionale Tumori IRCCS “Fondazione Pascale” Napoli Italy; ^10^ Department of Medical Oncology Centre Eugène Marquis Rennes France; ^11^ Division of Hematology‐Oncology, Department of Medicine, Samsung Medical Center Sungkyunkwan University School of Medicine Seoul Korea; ^12^ Product Development Oncology Genentech, Inc. South San Francisco CA USA

**Keywords:** breast cancer, circulating tumour DNA, *PIK3CA*, SANDPIPER, taselisib

## Abstract

Taselisib is a potent β‐sparing phosphatidylinositol 3‐kinase (PI3K) inhibitor that, with endocrine therapy, improves outcomes in phosphatidylinositol‐4,5‐bisphosphate 3‐kinase catalytic subunit alpha (*PIK3CA*)‐mutated (*PIK3CA*mut) advanced breast cancer. To understand alterations associated with response to PI3K inhibition, we analysed circulating tumour DNA (ctDNA) from participants enrolled in the SANDPIPER trial. Participants were designated as either *PIK3CA*mut or *PIK3CA* no mutation was detected (NMD) per baseline ctDNA. The top mutated genes and tumour fraction estimates identified were analysed for their association with outcomes. In participants with *PIK3CA*mut ctDNA treated with taselisib + fulvestrant, tumour protein p53 (*TP53*; encoding p53) and fibroblast growth factor receptor 1 (*FGFR1*) alterations were associated with shorter progression‐free survival (PFS) compared to participants with NMD in these genes. Conversely, participants with *PIK3CA*mut ctDNA harbouring a neurofibromin 1 (*NF1*) alteration or high baseline tumour fraction estimate experienced improved PFS upon treatment with taselisib + fulvestrant compared to placebo + fulvestrant. Broadly, alterations in oestrogen receptor (ER), PI3K and p53 pathway genes were associated with resistance to taselisib + fulvestrant in participants with *PIK3CA*mut ctDNA. Altogether, we demonstrated the impact of genomic (co‐)alterations on outcomes with one of the largest clinico‐genomic datasets of ER+, HER2−, *PIK3CA*mut breast cancer patients treated with a PI3K inhibitor.

## Graphical abstract

This study investigates the genomic alterations that are associated with response to PI3K inhibition and identifies mechanisms of resistance to PI3K inhibition using baseline and end‐of‐treatment circulating tumour DNA (ctDNA) from patients enrolled in SANDPIPER (NCT02340221). The SANDPIPER study generated one of the largest clinico‐genomic datasets of patients with ER+, HER2−, *PIK3CA*‐mutated breast cancer treated with a PI3K inhibitor.

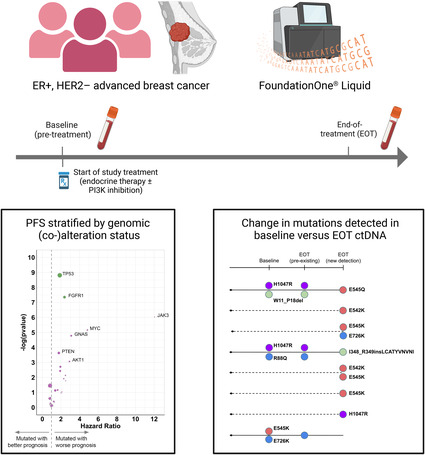

**Abbreviations**
AKT1: AKT serine/threonine kinase 1 (also known as protein kinase B)
ATM: Ataxia‐telangiesctasia mutated
BRAF: v‐raf murine sarcoma viral oncogene homolog B1
BRCA2: breast cancer gene 2
CAP: College of American Pathologists
CDH1: cadherin 1
CDK4/6: cyclin‐dependent kinase 4/6
CHEK2: checkpoint kinase 2
CI: confidence interval
CLIA: clinical laboratory improvement amendments
ctDNA: circulating tumour deoxyribonucleic acid
DDR2: discoidin domain receptor tyrosine kinase 2
EOT: end‐of‐treatment
ER(α): oestrogen receptor (alpha)
ESR1: oestrogen receptor 1
ET: endocrine therapy
F1L: FoundationOne^®^ Liquid
FGFR1: fibroblast growth factor receptor 1
FUL: fulvestrant
GNAS: guanine nucleotide‐binding protein, alpha stimulating
HER2: human epidermal growth factor receptor 2 (also known as ERBB2)
HR: Hazard ratio
HR+: hormone receptor‐positive
IQR: interquartile range
MAPK: mitogen‐activated protein kinase
mPFS: median progression‐free survival
MSAF: maximum somatic allele frequency
MYC: myelocytomatosis
NF1: neurofibromin 1
NGS: next‐generation sequencing
NMD: no mutation detected
p110α: phosphatidylinositol‐4,5‐bisphosphtate 3‐kinase catalytic subunit alpha protein, 110 kilodaltons
PALB2: partner and localizer of BRCA2
PBO: placebo
PCR: polymerase chain reaction
PFS: progression‐free survival
PI3K: phosphatidylinositol 3‐kinase
*PIK3CA*
: phosphatidylinositol‐4,5‐bisphosphate 3‐kinase catalytic subunit alpha
*PIK3CA*mut: *PIK3CA* mutated or mutation(s)
PTEN: phosphatase and tensin homolog
SNV: single‐nucleotide variant
TAS: taselisib
TFE: tumour fraction estimate
TP53: tumour protein p53

## 1 Introduction

The phosphatidylinositol 3‐kinase (PI3K) pathway is one of the most frequently dysregulated signalling pathways in human cancers and is involved in cell growth and proliferation. Activating mutations in *PIK3CA*, the gene that encodes the p110α catalytic subunit of the Class I PI3K enzyme, leads to dysregulation of PI3K signalling [1]. Across solid tumours, *PIK3CA* mutations (*PIK3CA*mut) are commonly observed in hormone receptor‐positive, HER2‐negative (HR+, HER2−) breast cancers, with a prevalence of ~ 40% [2, 3].

The clinical implementation of most PI3K inhibitors to date has been challenged by the toxicities induced by the inhibition of multiple p110 isoforms, particularly pan‐isoform inhibitors [4]. Taselisib is a potent and selective β‐sparing PI3K inhibitor [5] that was investigated in combination with fulvestrant in the phase III clinical trial, SANDPIPER, for patients with oestrogen receptor‐positive (ER+), HER2− locally advanced or metastatic breast cancer (NCT02340221) [6]. The study met its primary endpoint of improved progression‐free survival (PFS) with taselisib plus fulvestrant over placebo plus fulvestrant albeit with modest clinical activity (7.4 vs. 5.4 months; HR = 0.70) [6].

Circulating tumour DNA (ctDNA) is emerging as an important tool that appears representative of the DNA and the overall tumour mutational landscape of a patient's disease state [7, 8] and, for patients with ER+, HER2− advanced breast cancer, has prognostic and predictive response value [6, 9, 10]. Moreover, the low‐risk, non‐invasive procedure of a blood draw reduces the challenges associated with longitudinal sampling that exists for tumour biopsies. Ultimately, next‐generation sequencing of ctDNA leverages the relatively high concentrations of ctDNA detected in patients with advanced cancer [11] to enable studies that address questions about disease biology, tumour heterogeneity and mechanisms of resistance to targeted therapies, which were previously impractical because of the infeasibility of repeated tumour biopsies.

To investigate the potential alterations associated with response and/or resistance to endocrine therapy and/or PI3K inhibition in ER+, HER2− breast cancer tumours, we analysed baseline and end‐of‐treatment ctDNA collected from SANDPIPER participants.

## 2 Materials and methods

### 2.1 Study design and participants

The study design has been previously described [6]. Briefly, SANDPIPER was a randomized, double‐blind, placebo‐controlled, phase III trial evaluating the efficacy and safety of taselisib plus fulvestrant (TAS + FUL) versus placebo plus fulvestrant (PBO + FUL) in post‐menopausal women with ER+, HER2− locally advanced or metastatic breast cancer who had disease recurrence or progression during or after aromatase inhibitor therapy. Participants were randomized (2 : 1) to either the TAS + FUL or control (PBO + FUL) arm.

The SANDPIPER study protocol, including a description of the exploratory biomarker analyses of all randomized participants with and without *PIK3CA*mut tumours, was approved by the relevant Institutional Review Board/Ethics Committee at each participating centre prior to study initiation. The trial conformed to the Good Clinical Practice guidelines, the Declaration of Helsinki and applicable local laws. All participants provided signed informed consent, which included consent for the biomarker analyses included in this study.

### 2.2 Plasma ctDNA collection

Plasma samples were collected at different time points throughout the study. Herein, we present data from samples taken at baseline, defined as before the first dose of study treatment (pre‐dose at Cycle 1 Day 1) and at the end of study treatment (EOT), defined as the time‐point when a participant ceases the study treatment for reasons including but not limited to disease progression, toxicities, administrative reasons or at the investigator's discretion.

### 2.3 Comprehensive genomic profiling

Comprehensive genomic profiling of plasma samples using the FoundationOne^®^ Liquid (F1L) assay [12] was performed in a CLIA‐certified, CAP‐accredited laboratory (Foundation Medicine Inc., Cambridge, MA, USA) using hybrid‐capture, adapter ligation‐based libraries to identify genomic alterations (base substitutions, small insertions and deletions, copy number alterations and rearrangements/fusion events) for 70 cancer‐related genes. Processing of the sequence data and identification of different classes of genomic alterations were performed as previously described [13]. Unless otherwise indicated, the analysis is focused on alterations predicted to be pathogenic, defined as of known or likely oncogenic significance. Participants with *PIK3CA*mut were defined as those with ≥ 1 pathogenic single‐nucleotide variant in the *PIK3CA* gene detected in baseline ctDNA. Participants with *PIK3CA* no mutation detected (NMD) were defined as those without the detection of a pathogenic single‐nucleotide variant in the *PIK3CA* gene in baseline ctDNA.

Baseline plasma samples from 598 (94.8%) participants underwent genomic profiling, of which 508 (80.5%) samples were successfully sequenced and 90 samples (14.3%) failed processing. Baseline plasma samples were not collected from 33 (5.2%) participants due to withdrawal of consent or local regulatory testing restrictions. EOT plasma samples underwent genomic profiling for a subset of patients (15.8%; *n* = 100) who exhibited clinical benefit, defined as the best overall response by the investigator of partial response, complete response or stable disease with an extended PFS of > 7 months on study treatment with evidence of tumour shrinkage.

Quantification of the ctDNA fraction derived from tumour cells in blood/plasma samples was performed as previously described (unpublished data) [14] using two complementary methods: the proprietary tumour fraction estimator (TFE) and the maximum somatic allele frequency (MSAF) method. Tumour fraction estimator is based on a measure of tumour aneuploidy, and MSAF uses allele fraction from somatic coding alterations to estimate the ctDNA fraction. A high TFE was defined as ≥ 10% based on prior work that suggested 10% correlated with the proportion of tumour DNA adequate for high‐confidence copy number calls [15].

### 2.4 Statistical analyses

Statistical analysis, computation and plotting were performed using r version 3.6.1. Progression‐free survival data, from the clinical cut‐off date for the primary analysis (15 October 2017) [6], were analysed descriptively for each biomarker subgroup (mutated and NMD), and for each treatment arm (PBO + FUL and TAS + FUL). Kaplan–Meier survival analyses were performed with the log‐rank test using a Cox proportional hazards regression model to obtain hazard ratios and 95% confidence intervals. All statistical tests were two‐sided. To adjust for multiple comparisons, the Benjamini–Hochberg correction was used, wherein statistical significance was defined as a Benjamini–Hochberg‐adjusted *P*‐value (*q*‐value) < 0.05.

## 3 Results

### 3.1 Analysis population and participant demographics

As reported previously, 631 participants were enrolled and randomly assigned (2 : 1) to either the taselisib plus fulvestrant (TAS + FUL) or placebo plus fulvestrant (PBO + FUL) arm of SANDPIPER. For the clinical trial, participants were designated to either the *PIK3CA*mut or *PIK3CA* no mutation detected (NMD) cohort based on a centralized cobas^®^ *PIK3CA* Mutation Test result from formalin‐fixed paraffin‐embedded tissue collected prior to randomization, an eligibility criterion for all enrolled participants [6]. However, for the analysis herein, *PIK3CA* mutation status was solely based on results from baseline plasma using the F1L assay. Of note, the majority of participants in SANDPIPER (97.0%) were cyclin‐dependent kinase 4/6 (CDK4/6) inhibitor‐naïve given that they were randomized between 2015 and 2017 [6], which coincided with the initial approvals of CDK4/6 inhibitors for HR+, HER2− breast cancer treatment.

**Fig. 1:**
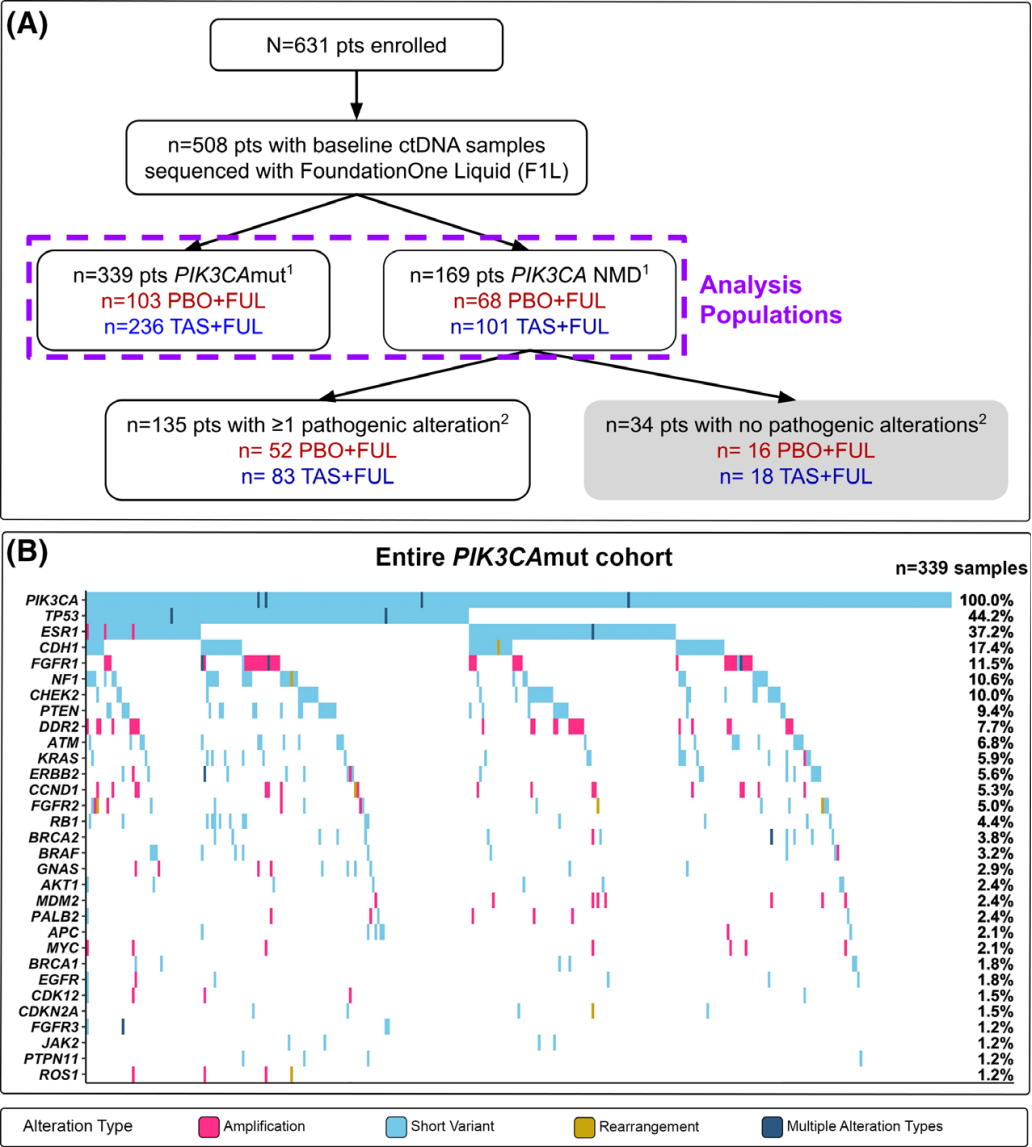
Analysis of baseline ctDNA from participants with ER+, HER2− advanced breast cancer. (A) Consort diagram for baseline circulating tumour DNA (ctDNA) analysis of participants enrolled in the SANDPIPER study. ^1^ *PIK3CA* mutation status is determined by detection of ≥ 1 pathogenic *PIK3CA* mutation in baseline ctDNA. ^2^Stratification is based on the detection of ≥ 1 pathogenic alteration in baseline ctDNA. (B) Genomic landscape of baseline ctDNA from participants with *PIK3CA*mut, ER+, HER2− advanced breast cancer. Only genes that were altered in ≥ 1% of samples (*n* ≥ 3) are shown in the tile plot. Individual samples may harbour ≥ 1 alterations of the same variant type in a single gene (e.g. ≥ 1 *TP53* short variants); this information is not denoted in the tile plot. ER, oestrogen receptor; F1L, FoundationOne Liquid; FUL, fulvestrant; HER2, human epidermal growth factor receptor 2; mut, mutated; *n*, sample size; PBO, placebo; pts, patients; TAS, taselisib.

For the analyses herein (Fig. 1A), *PIK3CA* mutation status (*PIK3CA*mut or *PIK3CA* NMD) was based on the comprehensive genomic profiling (CGP) of baseline plasma samples using the F1L assay. In the 508 (80.5%) participants for whom *PIK3CA* mutation status is known from both the tissue‐based cobas^®^ *PIK3CA* Mutation Test and the baseline ctDNA‐based F1L assay, the sensitivity is 78.2% and the specificity is 86.7% [6]. These metrics may be reflective of the methodological differences in the assays (PCR vs. NGS) and the temporal differences in when the samples were collected; specifically, plasma samples were freshly collected during the study screening period, whereas tumour tissue samples were largely archival samples, many from the time of primary diagnosis. Of the 339 participants with tumours classified as ctDNA *PIK3CA*mut, 103 (30.4%) and 236 (69.6%) were treated with PBO + FUL and TAS + FUL respectively. Of the 169 participants with tumours classified as ctDNA *PIK3CA* NMD, 68 (40.2%) and 101 (59.8%) were treated with PBO + FUL and TAS + FUL, respectively. Demographics and disease characteristics were largely similar between participants with *PIK3CA*mut and *PIK3CA* NMD ctDNA between the treatment arms (Table S1), which was consistent with the total SANDPIPER population [6]. Of note, bone metastases (inclusive of participants with and without bone‐only disease) were found to occur at a higher frequency in participants with pathogenic alterations detected in baseline ctDNA than in participants with no pathogenic alterations detected in baseline ctDNA [77.8% (*n* = 369/474) vs. 38.2% (*n* = 13/34); *P* < 0.001, Fisher's Exact Test].

### 3.2 Genomic landscape of baseline ctDNA

Amongst the 339 participants with *PIK3CA*mut baseline ctDNA, the baseline ctDNA of 20.6% (*n* = 70) harboured ≥ 2 *PIK3CA* alterations and 19.5% (*n* = 66) harboured ≥ 2 *PIK3CA* single‐nucleotide variants (SNVs). Of the cumulative 428 *PIK3CA* alterations detected in baseline ctDNA from the *PIK3CA*mut participants across both study arms, 0.9% (*n* = 4) were copy number amplifications and 99.1% (*n* = 424; Fig. S1, Table S2) were short variants, comprised largely of well‐defined ‘hotspot mutations’ in the *PIK3CA* gene [i.e. occurring at amino acids H1047 (34.1%; *n* = 146/428), E545 (25.0%; *n* = 107/428) and E542 (12.6%; *n* = 54/428)].

Amongst the 339 participants with *PIK3CA*mut baseline ctDNA, the top genes co‐altered with *PIK3CA* were *TP53* (44.2%), *ESR1* (37.2%), *CDH1* (17.4%), *FGFR1* (11.5%), *NF1* (10.6%), *CHEK2* (10.0%) and *PTEN* (9.4%) (Fig. 1B). Amongst the 169 participants with *PIK3CA* NMD baseline ctDNA, the top altered genes were *TP53* (32.0%), *ESR1* (28.4%), *CDH1* (16.0%) and *ATM* (10.1%) (Fig. S2C). Within each of the *PIK3CA*mut and *PIK3CA* NMD cohorts, no statistically significant difference was observed in the frequency of altered genes between treatment arms (*q* > 0.05, Fisher's Exact Test; Fig. S2A,B,D,E). Between the *PIK3CA*mut and *PIK3CA* NMD cohorts, the frequency of *DDR2* alterations was significantly higher in the *PIK3CA*mut cohort [7.7% (*n* = 26/339) vs. 1.2% (*n* = 2/169); *q* = 0.050] (Fig. 1B, Fig. S2C). No other genes were significantly differentially altered between the two cohorts (*q* > 0.05, Fisher's Exact Test). However, between the *PIK3CA*mut and *PIK3CA* NMD cohorts, there was a trend towards an increased frequency in the *PIK3CA*mut cohort of alterations of the following genes: *TP53* [44.2% (*n* = 150/339) vs. 32.0% (*n* = 54/169); *P* = 0.0094; *q* = 0.20], *PTEN* [9.4% (*n* = 32/339) vs. 4.1% (*n* = 7/169); *P* = 0.035; *q* = 0.53] and *NF1* [10.6% (*n* = 36/339) vs. 5.3% (*n* = 9/169); *P* = 0.048; *q* = 0.53].

### 3.3 Genomic analysis of baseline ctDNA and association with clinical outcomes

To evaluate whether any of the top altered genes in the participants' baseline ctDNA correlated with prognosis or treatment outcomes, we next analysed the association between genomic alteration status and PFS within each of the treatment arms. Consistent with the previously reported hazard ratios (HRs) [6], the PFS HR based on *PIK3CA* ctDNA status was 0.65 between treatment arms in the entire *PIK3CA*mut cohort and 0.85 between treatment arms for the entire *PIK3CA* NMD cohort.

#### 3.3.1 *PIK3CA*mut cohort

**Fig. 2:**
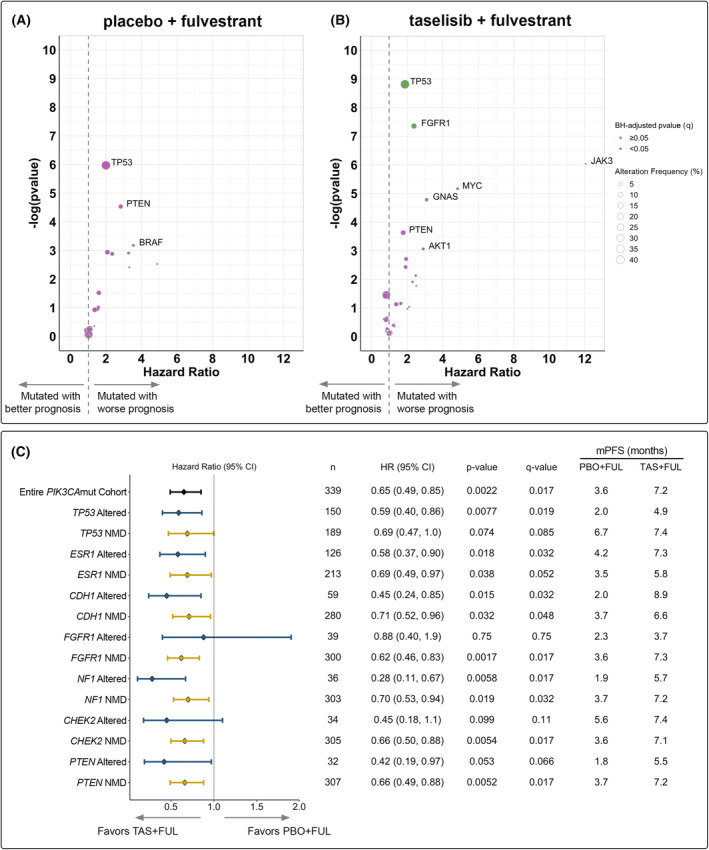
Association of progression‐free survival (PFS) with (A, B) genomic alteration status and (C) study treatment in participants with *PIK3CA*mut baseline ctDNA. Association between PFS and genomic alteration status in (A) PBO + FUL‐treated participants and (B) TAS + FUL‐treated participants. Only genes that were altered in ≥ 1% of samples (*n* ≥ 3) are shown in the volcano plots; the gene is annotated if the nominal *P*‐value < 0.05. The size of the bubble indicates the frequency of the alterations in the respective gene within the treatment arm. (C) PFS by biomarker status of the top altered genes (altered in ≥ 10% of samples). Log‐rank tests using a Cox proportional hazards regression model were used to obtain hazard ratio (HR) and *P*‐values; statistical significance is defined by Benjamini–Hochberg‐adjusted *P*‐value (*q*‐value) < 0.05. CI, confidence interval; FUL, fulvestrant; mPFS, median progression‐free survival; mut, mutated; *n*, sample size; PBO, placebo; TAS, taselisib.

**Fig. 3:**
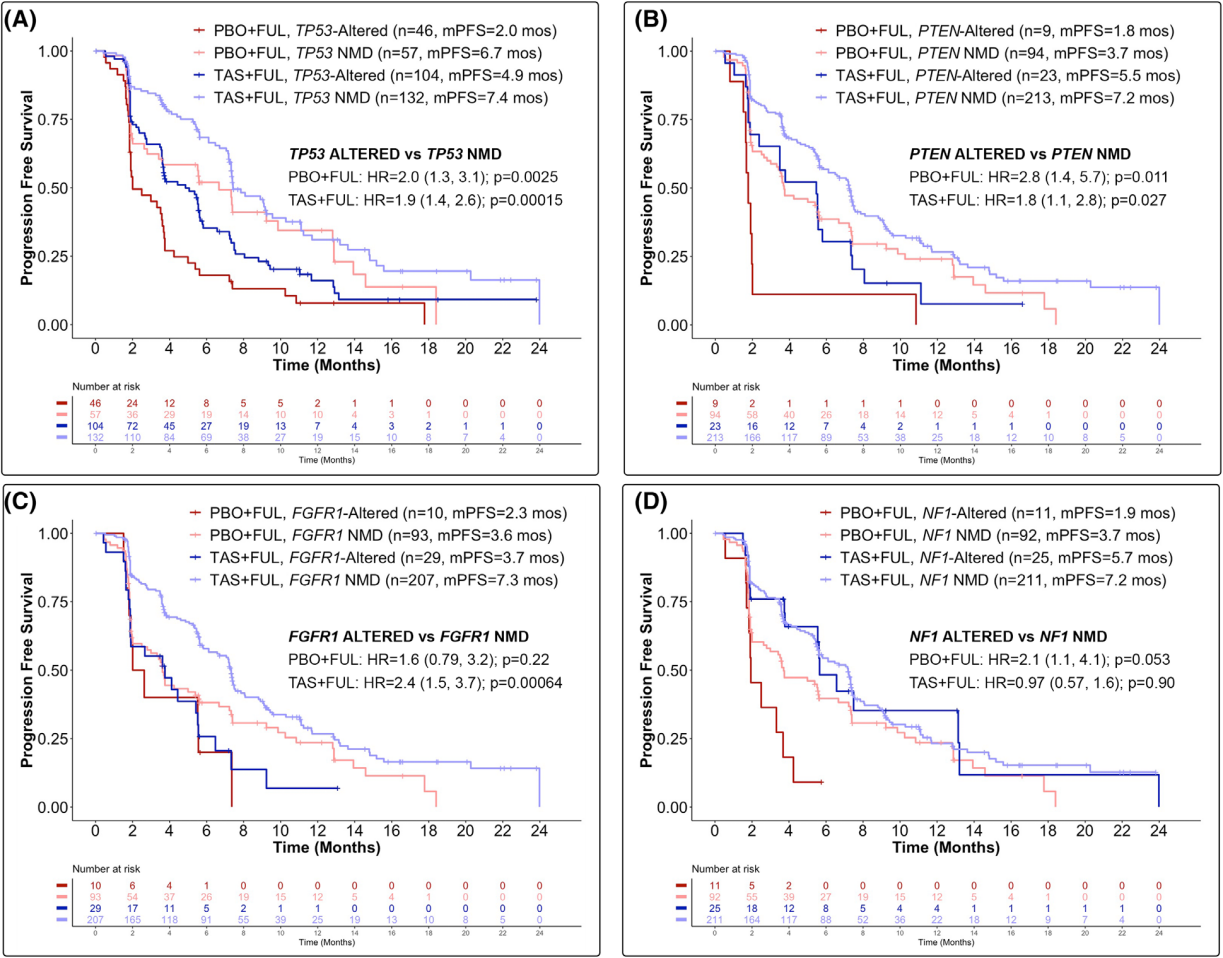
Kaplan–Meier plots of progression‐free survival (PFS) by treatment arm and alteration status for the *PIK3CA*mut cohort. PFS by detectable (A) *TP53*, (B) *PTEN*, (C) *FGFR1* and (D) *NF1* alteration(s) in baseline ctDNA for *PIK3CA*mut participants in the PBO + FUL and TAS + FUL arms. Tick marks indicate censoring events. Log‐rank tests using a Cox proportional hazards regression model were used to obtain hazard ratio (HR) and *P*‐values. FGFR1, fibroblast growth factor receptor 1; FUL, fulvestrant; mPFS, median progression‐free survival; mut, mutated; mos, months; *n*, sample size; NF1, neurofibromin 1; NMD, no mutation detected; PBO, placebo; PTEN, Phosphatase and tensin homolog; TAS, taselisib; TP53, tumour protein p53.

In participants treated with PBO + FUL, alterations in *TP53* [median PFS (mPFS): 2.0 vs. 6.7 months; HR = 2.0 (95% confidence interval (CI) 1.3–3.1); *P* = 0.0025; *q* = 0.069], *PTEN* [mPFS: 1.8 vs. 3.7 months; HR = 2.8 (95% CI 1.4–5.7); *P* = 0.011; *q* = 0.14] and *BRAF* [mPFS: 1.8 vs. 3.7 months; HR = 3.5 (95% CI 1.3–9.9); *P* = 0.042; *q* = 0.35] trended towards shorter PFS compared to participants with NMD in these genes (Figs 2A and 3A,B, Fig. S3A). In participants treated with TAS + FUL, alterations in *TP53* [mPFS: 4.9 vs. 7.4 months; HR = 1.9, 95% CI 1.4–2.6; *q* = 0.016] and *FGFR1* [mPFS: 3.7 vs. 7.3 months; HR = 2.4, 95% CI 1.5–3.7; *q* = 0.035] were significantly associated with shorter PFS compared to participants with NMD in these genes (Figs 2B and 3A,C, Fig. S3A). Furthermore, in participants treated with TAS + FUL, alterations in *PTEN* [mPFS: 5.5 vs. 7.2 months; HR = 1.8 (95% CI 1.1–2.8); *P* = 0.027; *q* = 0.26], *AKT1* [mPFS: 3.2 vs. 7.2 months; HR = 2.9 (95% CI 1.2–7.2); *P* = 0.047; *q* = 0.35], *GNAS* [mPFS: 2.8 vs. 7.2 months; HR = 3.1 (95% CI 1.5–6.3); *P* = 0.0084; *q* = 0.13] and *MYC* [mPFS: 1.9 vs. 7.2 months; HR = 4.8 (95% CI 2.0–12); *P* = 0.0057; *q* = 0.10] trended towards shorter PFS compared to participants with NMD in these genes (Figs 2B and 3B, Fig. S3A). Of the other top genes co‐altered with *PIK3CA*, alterations in *ESR1*, *CDH1*, *NF1* or *CHEK2* were not associated with differential PFS outcomes in either treatment arm (Fig. 2A,B, Fig. S3A). Furthermore, a largely consistent PFS benefit with TAS + FUL was observed, independent of the genomic alteration status, with HR point estimates < 1.0 for all subgroups assessed (Fig. 2C). Interestingly, we observed a significantly longer PFS in participants with *NF1*‐altered ctDNA treated with TAS + FUL compared to those treated with PBO + FUL [mPFS: 5.7 vs. 1.9 months; HR = 0.28, 95% CI 0.11–0.67; *q* = 0.017] (Figs 2C and 3D).

#### 3.3.2 *PIK3CA* NMD cohort

In participants treated with PBO + FUL, alterations in *TP53* [mPFS: 3.0 vs. 7.5 months; HR = 2.6, 95% CI 1.4–4.9; *P* = 0.0062; *q* = 0.28], *CDH1* [mPFS: 3.1 vs. 7.3 months; HR = 2.7, 95% CI 1.3–5.4; *P* = 0.012; *q* = 0.28], *ESR1* [mPFS: 4.7 vs. 7.3 months; HR = 2.1, 95% CI 1.1–3.9; *P* = 0.022; *q* = 0.28] and *ERBB2* [mPFS: 2.0 vs. 6.9 months; HR = 5.3 (95% CI 1.5–18); *P* = 0.029; *q* = 0.28] trended towards shorter PFS compared to participants with NMD in these genes (Figs S3B, S4A, S5A–C). In participants treated with TAS + FUL, alterations in *ESR1* [mPFS: 4.6 vs. 9.1 months; HR = 1.8, 95% CI 1.1–3.0; *P* = 0.020; *q* = 0.28], *BRAF* [mPFS: 2.0 vs. 8.9 months; HR = 5.5 (95% CI 1.6–18); *P* = 0.025; *q* = 0.28] and *PALB2* [mPFS: 3.6 vs. 8.9 months; HR = 1.7 (95% CI 1.7–19); *P* = 0.022; *q* = 0.28] trended towards shorter PFS compared to participants with NMD in these genes (Figs S3B, S4B, S5C). *ATM* alterations were not associated with differential PFS outcomes in either treatment arm (Figs S3B and S5D). Additionally, we observed a significantly longer PFS in participants with either *TP53*‐altered ctDNA [mPFS: 9.1 vs. 3.0 months; HR = 0.35, 95% CI 0.18–0.70; *q* = 0.025] or *CDH1*‐altered ctDNA [mPFS: 11.1 vs. 3.1 months; HR = 0.21, 95% CI 0.065–0.69; *q* = 0.025] treated with TAS + FUL compared to those treated with PBO + FUL (Figs S4C and S5A,B).

### 3.4 Baseline tumour fraction estimate and clinical outcomes

Recent studies have reported that a larger treatment effect is observed in ER+, HER2− advanced breast cancer patients treated with ET when outcomes are analysed by *PIK3CA*mut positivity in ctDNA compared to the same analysis by tissue *PIK3CA*mut positivity [6, 10, 16]. Here, we further dissected the ctDNA‐positive population using the baseline tumour fraction estimate (TFE), a measure of the quantity of ctDNA shed from the tumour into circulation.

#### 3.4.1 *PIK3CA*mut cohort

**Fig. 4:**
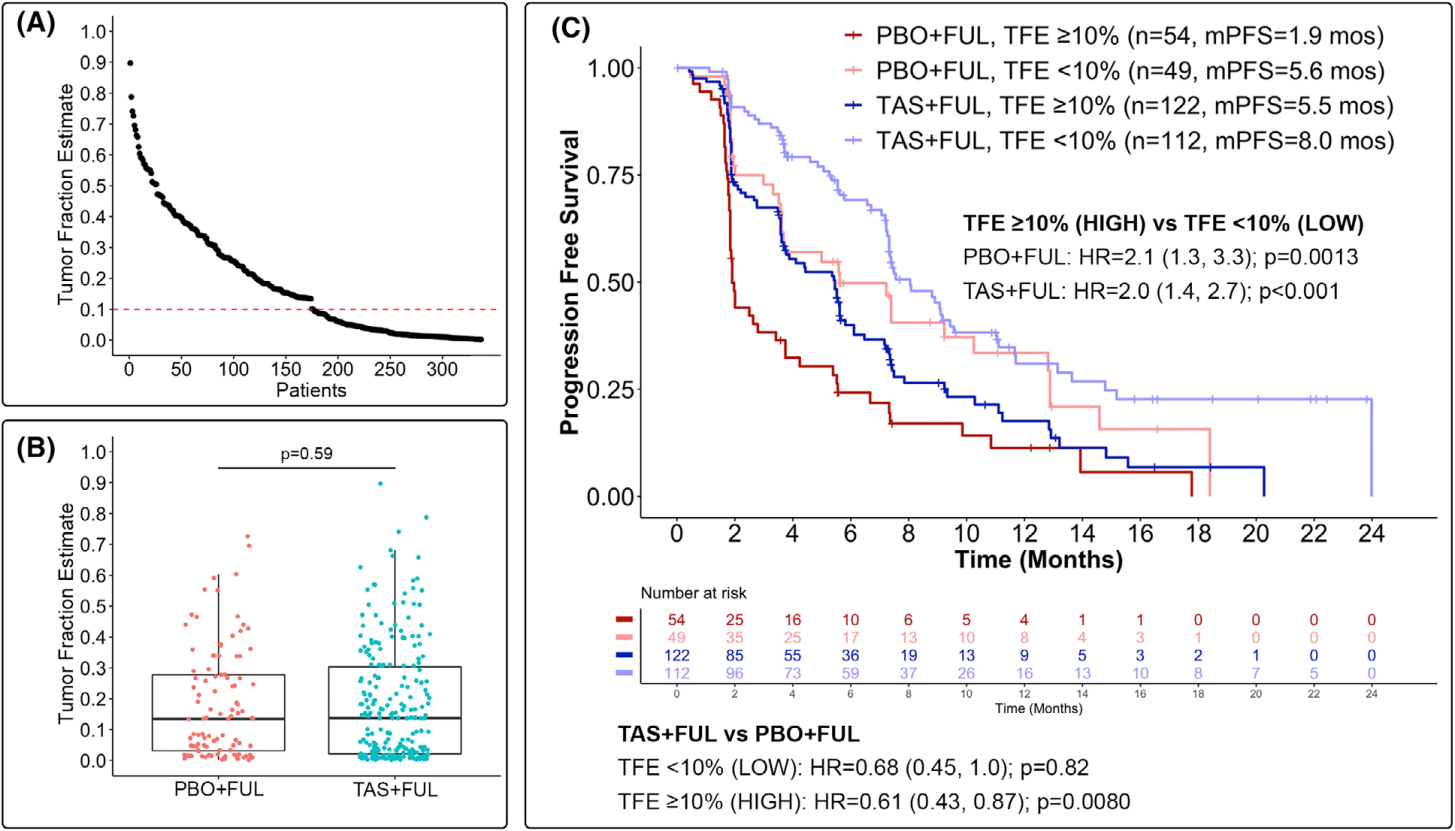
Association between tumour fraction estimate (TFE) and progression‐free survival (PFS) in participants with *PIK3CA*mut baseline ctDNA. (A) Distribution of TFE at baseline. Red dashed line (TFE = 0.1) denotes cut‐off for high TFE (≥ 10%) versus low TFE (< 10%). (B) Distribution of TFE at baseline stratified by study treatment. Amongst TAS + FUL‐treated participants, the median TFE was 0.14 (IQR 0.02–0.30); amongst PBO + FUL‐treated participants, the median TFE was 0.14 (IQR 0.03–0.28). *P*‐value obtained from Wilcoxon rank‐sum test. (C) Association between TFE at baseline and PFS. Log‐rank tests using a Cox proportional hazards regression model were used to obtain hazard ratio (HR) and *P*‐values. FUL, fulvestrant; IQR, interquartile range; mPFS, median progression‐free survival; mut, mutated; mos, months; PBO, placebo; TAS, taselisib.

Within the *PIK3CA*mut cohort, high baseline TFE was observed in 176 participants, of whom 54 (30.7%) and 122 (69.3%) were treated with PBO + FUL and TAS + FUL, respectively; low baseline TFE was observed in 161 participants, of whom 49 (30.4%) and 112 (69.6%) were treated with PBO + FUL and TAS + FUL respectively (Fig. 4A). Of the 339 baseline samples evaluated, the tumour fractions were inestimable for two samples. No statistically significant difference was observed in the median baseline TFE between treatment arms (TAS + FUL median TFE = 0.14 vs. PBO + FUL median TFE = 0.14; *P* = 0.59, Wilcoxon rank‐sum test; Fig. 4B). Moreover, a strong correlation was observed between TFE and *PIK3CA*mut allele fraction in baseline ctDNA (Pearson's correlation *r* = 0.82, *P* < 0.001), suggesting that *PIK3CA* mutations were predominantly clonal mutations rather than passenger mutations. Median PFS was shorter for participants with high baseline TFE compared to those with low baseline TFE within the PBO + FUL (mPFS: 1.9 vs. 5.6 months; HR = 2.1, 95% CI 1.3–3.3; *q* = 0.0032) and TAS + FUL (mPFS: 5.5 vs. 8.0 months; HR = 2.0, 95% CI 1.4–2.7; *q* < 0.001) treatment arms (Fig. 4C). Furthermore, of participants with high baseline TFE, those who received TAS + FUL experienced longer PFS compared to those who received PBO + FUL [mPFS: 5.5 vs. 1.9 months; HR = 0.61 (0.43, 0.87); *q* = 0.013]. For participants with low baseline TFE, despite a similar observed HR, the longer PFS experienced by participants treated with TAS + FUL compared to PBO + FUL was not statistically significant [mPFS: 8.0 vs. 5.6 months; HR = 0.68 (0.45, 1.0); *q* = 0.82].

#### 3.4.2 *PIK3CA* NMD cohort

Within the *PIK3CA* NMD cohort, high baseline TFE was observed in 52 participants and low baseline TFE was observed in 109 participants (Fig. S6A). Of the 169 baseline samples evaluated, the tumour fractions were inestimable for eight samples. With both treatment arms combined, the median baseline TFE for the *PIK3CA* NMD cohort was lower than that of the *PIK3CA*mut cohort (*PIK3CA*mut median TFE = 0.14 vs. *PIK3CA* NMD median TFE = 0.02; *P* < 0.001, Wilcoxon rank‐sum test; Fig. S6B). Furthermore, no statistically significant difference was observed in the median baseline TFE between treatment arms (TAS + FUL median TFE = 0.01 vs. PBO + FUL median TFE = 0.02; *P* = 0.50, Wilcoxon rank‐sum test; Fig. S6C). Similar to the *PIK3CA*mut cohort, participants with high baseline TFE experienced worse PFS than participants with low baseline TFE within both the PBO + FUL [mPFS: 3.0 vs. 7.3 months; HR = 3.0, 95% CI 1.6–5.6; *q* = 0.0015] and TAS + FUL [mPFS: 3.6 vs. 9.4 months; HR = 3.1, 95% CI 1.9–5.0; *q* < 0.001] treatment arms (Fig. S6D). In contrast to the *PIK3CA*mut cohort, however, no difference in PFS was observed between TAS + FUL‐treated participants compared to PBO + FUL‐treated participants with either high baseline TFE [mPFS: 3.6 vs. 3.0 months; HR = 0.82, 95% CI 0.45–1.5; *q* = 0.54] or low baseline TFE [9.4 vs. 7.3 months; HR = 0.86, 95% CI 0.53–1.4; *q* = 0.54].

### 3.5 Genomic landscape of EOT ctDNA in participants who experienced clinical benefit from study treatment

**Fig. 5:**
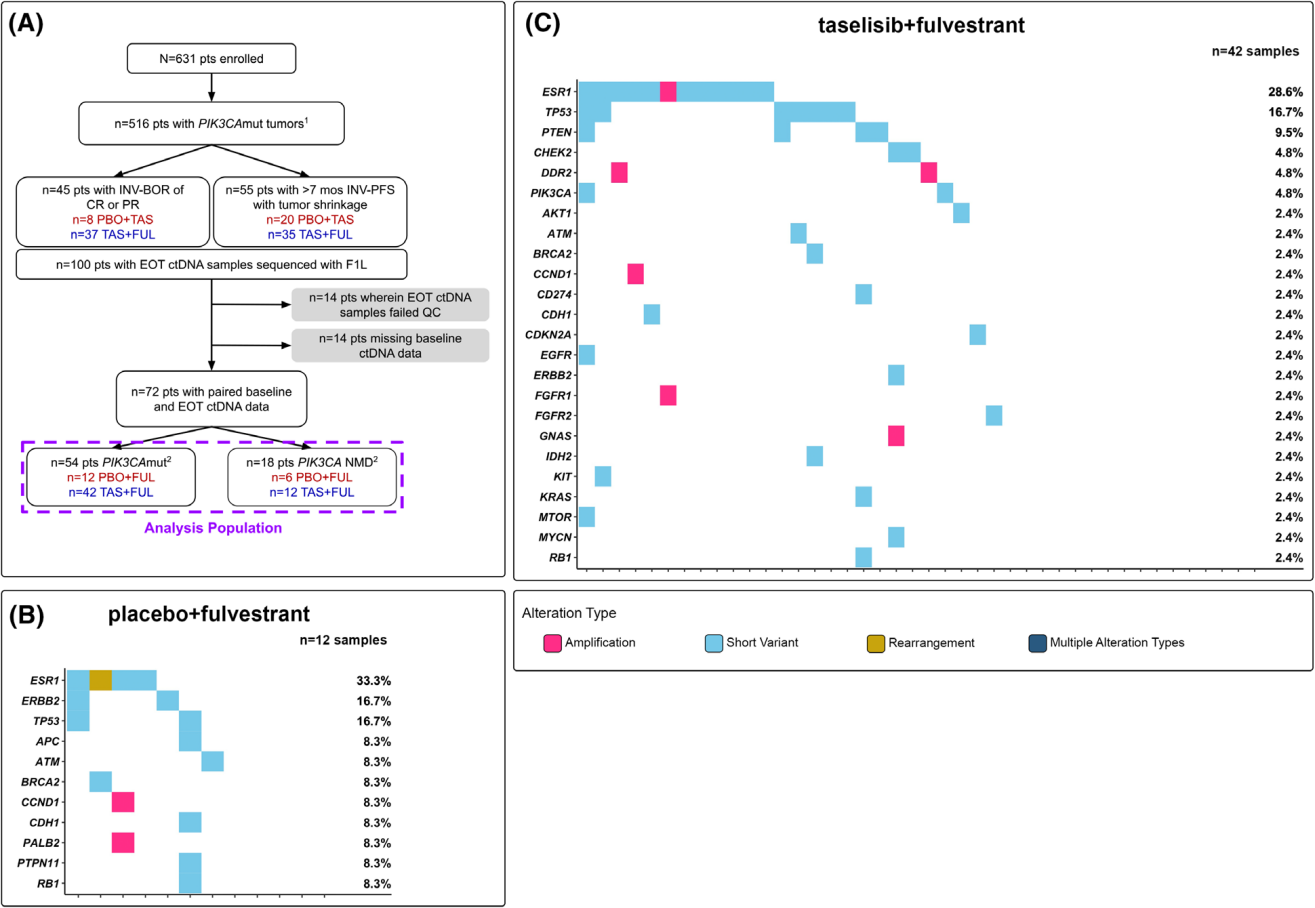
Paired analysis of baseline and EOT ctDNA from participants enrolled in the SANDPIPER study who exhibited clinical benefit. (A) Consort diagram of the analysis population. Genomic landscape of newly detected alterations at EOT in participants with *PIK3CA*mut baseline ctDNA treated with (B) PBO + FUL and (C) TAS + FUL. Newly detected alterations are defined as alterations that were not detected in baseline ctDNA but were detected in EOT ctDNA. ^1^ *PIK3CA* mutation status is determined by cobas^®^ *PIK3CA* Mutation testing of baseline tumour tissue. ^2^ *PIK3CA* mutation status is determined by detection of ≥ 1 pathogenic *PIK3CA* mutation in baseline ctDNA. CR, complete response; ctDNA, circulating tumour DNA; EOT, end‐of‐treatment; FUL, fulvestrant; INV‐BOR, investigator‐assessed best overall response; INV‐PFS, investigator‐assessed progression‐free survival; mut, mutated; mos, months; *n*, sample size; NMD, no mutation detected; PBO, placebo; PR, partial response; pts, patients; QC, quality control; TAS, taselisib.

End‐of‐treatment (EOT) plasma samples were submitted for F1L genomic profiling from 100 participants with *PIK3CA*mut tumours who exhibited clinical benefit. F1L data were evaluable for both the baseline and EOT ctDNA samples for 72 of these 100 participants. Of the 72 participants with paired evaluable ctDNA, 54 of those participants' baseline ctDNA harboured a *PIK3CA*mut, of which 12 (22.2%) were treated with PBO + FUL and 42 (77.8%) were treated with TAS + FUL; 18 of those participants' baseline ctDNA were *PIK3CA* NMD, of which 6 (33.3%) were treated with PBO + FUL and 12 (66.7%) were treated with TAS + FUL (Fig. 5A).

We investigated the spectrum of alterations detected in EOT ctDNA from the participants for whom paired baseline and EOT ctDNA data were available. Similar to baseline ctDNA, all *PIK3CA* alterations detected in EOT ctDNA across both study arms were short variants, specifically, SNVs (*n* = 65 in *PIK3CA*mut participants; *n* = 11 in *PIK3CA* NMD participants), comprised largely of hotspot mutations in *PIK3CA* [i.e. occurring at amino acids H1047 (26.3%; *n* = 20), E545 (22.4%; *n* = 17) and E542 (17.1%; *n* = 13)]. Amongst the eight *PIK3CA* NMD participants with a detectable *PIK3CA* SNV at EOT, three participants each harboured two *PIK3CA* SNVs and five participants each harboured a single *PIK3CA* SNV. Of note, while these eight *PIK3CA* NMD participants did not have any *PIK3CA* SNV detected in baseline ctDNA, they each had a detectable alteration in at least one of the 69 other cancer‐related genes evaluated on the F1L assay, suggesting that ctDNA was present in the blood of these participants. Amongst the *PIK3CA*mut participants (*n* = 12 PBO + FUL‐treated; *n* = 42 TAS + FUL‐treated), the top altered genes at EOT upon PBO + FUL treatment were *PIK3CA* (100%), *ESR1* (66.7%), *TP53* (33.3%), *CDH1* (25.0%), *ATM* (16.7%), *BRCA2* (16.7%), *CHEK2* (16.7%) and *ERBB2* (16.7%); the top altered genes at EOT upon TAS + FUL treatment were *PIK3CA* (92.9%), *ESR1* (42.9%), *TP53* (31.0%), *CDH1* (21.4%), *CHEK2* (14.3%) and *PTEN* (11.9%) (Fig. S7A,B). Amongst *PIK3CA* NMD participants (*n* = 6 PBO + FUL‐treated; *n* = 12 TAS + FUL‐treated), the altered genes at EOT upon PBO + FUL treatment were *PIK3CA* (50.0%), *CDH1* (33.3%), *ATM* (16.7%), *CHEK2* (16.7%), *ESR1* (16.7%) and *TP53* (16.7%); the top altered genes at EOT upon TAS + FUL treatment were *TP53* (50.0%), *PIK3CA* (41.7%), *ESR1* (33.3%), *ATM* (16.7%), *CDH1* (16.7%), *CHEK2* (16.7%), *FGFR1* (16.7%) and *NF1* (16.7%) (Fig. S8A,B). Within each of the *PIK3CA*mut and *PIK3CA* NMD cohorts, no statistically significant difference was observed in the frequency of altered genes between the treatment arms (*q* > 0.05, Fisher's Exact Test).

### 3.6 Paired baseline and EOT ctDNA analysis of *PIK3CA*mut participants who experienced clinical benefit from study treatment

**Fig. 6:**
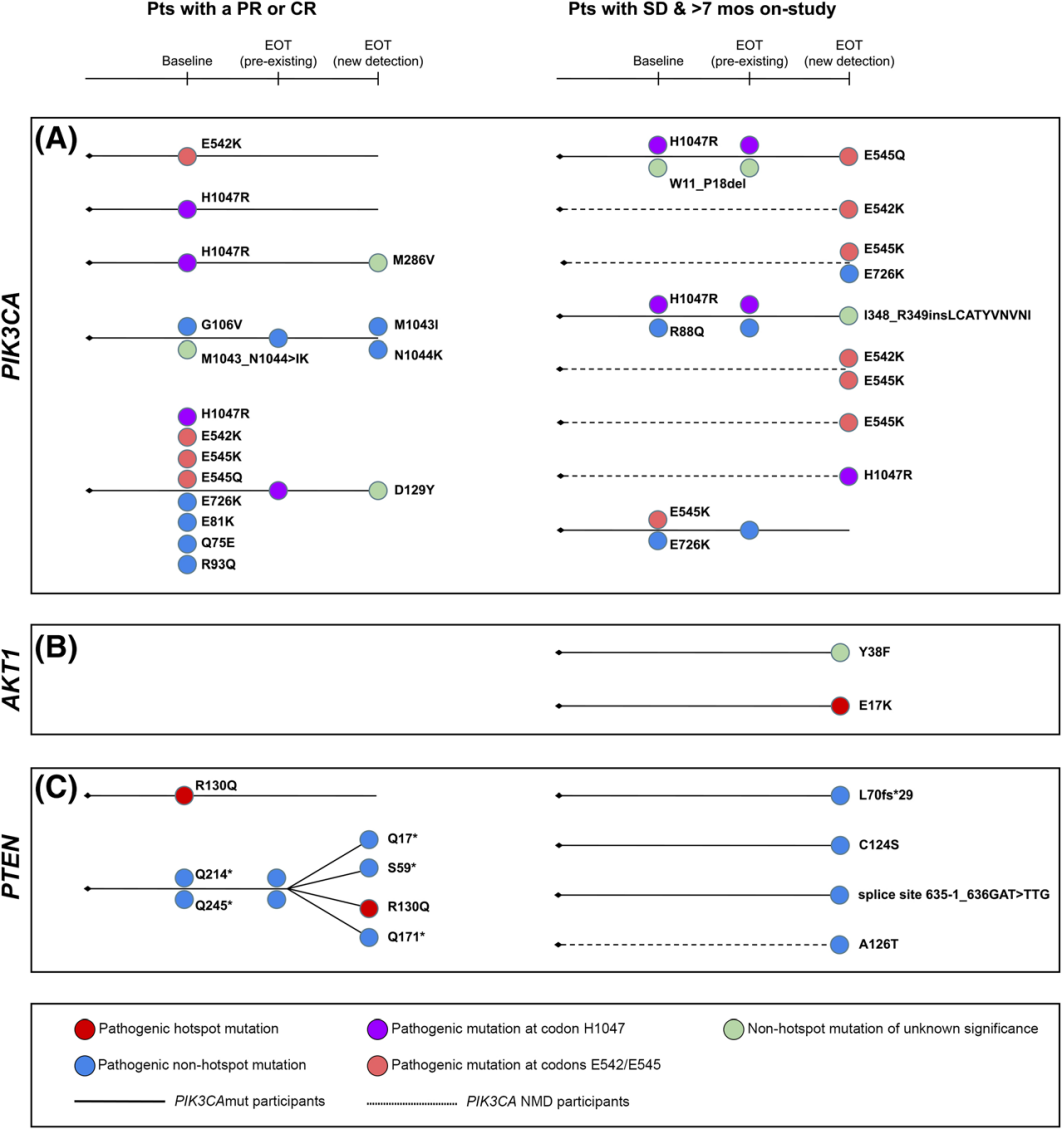
Circulating tumour DNA (ctDNA) dynamics of PI3K pathway genes in TAS + FUL‐treated participants who exhibited clinical benefit. ctDNA dynamics of (A) *PIK3CA*, (B) *AKT1* and (C) *PTEN* mutations in end‐of‐treatment (EOT) ctDNA compared to baseline ctDNA. Sample shown only if a change in mutation(s) detected was observed between baseline and EOT ctDNA. Dendrogram includes short variants that were classified as either pathogenic (i.e. functionally relevant) or variants of unknown significance. Samples on the left are from participants with the best overall response by investigator of partial response (PR) or complete response (CR) to TAS + FUL treatment; samples on the right are from participants with stable disease (SD) in response to TAS + FUL treatment who demonstrated extended PFS of > 7 months with tumour shrinkage. AKT1, AKT serine/threonine kinase 1 (also known as protein kinase B); FUL, fulvestrant; mos, months; mut, mutated; NMD, no mutation detected; PBO, placebo; PI3K, phosphatidylinositol 3‐kinases; PTEN, Phosphatase and tensin homolog; TAS, taselisib.

To investigate the impact of the study treatment on the genomic landscape of EOT ctDNA in participants who experienced clinical benefit, we compared the genomic alterations that were detected in EOT ctDNA but not detected in baseline ctDNA. Amongst the *PIK3CA*mut participants, the top newly detected alterations upon PBO + FUL treatment were in *ESR1* (33.3%), *ERBB2* (16.7%) and *TP53* (16.7%); the top newly detected alterations upon TAS + FUL treatment were in *ESR1* (28.6%), *TP53* (16.7%) and *PTEN* (9.5%) (Fig. 5B,C and Fig. S7C). Amongst the *PIK3CA* NMD participants, the newly detected alterations upon PBO + FUL treatment were in *PIK3CA* (50.0%), *CDH1* (16.7%), *CHEK2* (16.7%) and *ESR1* (16.7%); the top newly detected alterations upon TAS + FUL treatment were in *PIK3CA* (41.7%), *ESR1* (33.3%), *ATM* (16.7%), *CDH1* (16.7%), *CHEK2* (16.7%) and *FGFR1* (16.7%) (Fig. S8C,D). Within each of the *PIK3CA*mut and *PIK3CA* NMD cohorts, no statistically significant difference was observed in the alteration rates of individual genes newly detected at EOT between the treatment arms (*q* > 0.05, Fisher's Exact Test). Lastly, to study the scope of newly detected mutations specifically in PI3K pathway genes, we assessed the changes in *PIK3CA*, *AKT1* and *PTEN* detected in EOT ctDNA compared to baseline ctDNA in TAS + FUL‐treated participants. We observed the detection of two new *AKT1* mutations, eight new *PTEN* mutations and 10 unique new *PIK3CA* mutations (Fig. 6A–C). Of note, each of the participants whose EOT ctDNA harboured these newly detected mutations in the PI3K pathway genes was found to also have additional non‐PI3K pathway genomic alterations.

## 4 Discussion

As the SANDPIPER population had received prior aromatase inhibitor therapy and was predominantly CDK4/6‐inhibitor naïve, the genomic landscape of baseline ctDNA was expected to reflect that of an endocrine‐resistant population. In both the *PIK3CA* mut and *PIK3CA* NMD cohorts, the top altered genes detected in baseline ctDNA included genes associated with the ER (*ESR1*), PI3K (*PIK3CA*, *PTEN*), p53 (*TP53*, *ATM*, *CHEK2*), MAPK (*NF1*), and/or receptor tyrosine kinase (*FGFR1*) signalling pathways (Fig. 1B and Fig. S2C). These observations are consistent with the molecular profiles of endocrine‐resistant ER+, HER2− breast cancer tumours [3, 17, 18]. Resistance resulting from *ESR1* mutations has been well‐characterized in hormone‐refractory disease [19, 20] and has been shown to correlate with worse outcomes following ET [21, 22]. Activation of PI3K signalling through mutations in *PIK3CA*, *AKT1* or *PTEN* has been shown to confer endocrine resistance *in vitro* [23, 24]. Moreover, *in vitro* data have shown that increased MAPK pathway signalling can promote the loss of ERα expression in breast cancer tumours [25], which may contribute to the poor responses to ET observed in patients whose tumours harbour alterations in genes associated with the MAPK pathway [3]. Of note, the prevalence of *TP53* alterations detected in baseline ctDNA was higher than that reported in another analysis of advanced HR+, HER2− breast cancer tumour tissue [3] and may be due to the inclusion of *TP53* somatic mutations that occur in clonal haematopoiesis, which is detectable in peripheral blood [26].

To expand our understanding of ER+, HER2− breast cancer disease biology, we investigated the association of genomic alterations with PFS to identify patient populations who may be intrinsically resistant to study treatment. Our analysis showed that in PBO + FUL‐treated participants with *PIK3CA*mut baseline ctDNA, alterations in *TP53*, *PTEN* and *BRAF* trended towards poor prognosis. In TAS + FUL‐treated participants with *PIK3CA*mut baseline ctDNA, alterations in *TP53* and *FGFR1* were associated with poor prognosis, and alterations in *PTEN*, *AKT1*, *GNAS* and *MYC* trended towards poor prognosis. Numerous studies have demonstrated that *TP53* alterations confer clinical resistance to ET [17, 27, 28, 29], and *in vitro* studies have suggested that *TP53* mutations may confer resistance to PI3K inhibition [30]. Furthermore, the loss of the tumour suppressor PTEN, a negative regulator of the PI3K pathway, is associated with clinical resistance to ET [31] and p110α inhibition [32, 33]. Mutations in *PIK3CA* and *PTEN* have been described to be largely mutually exclusive in breast cancers [34]; therefore, we speculated that the detection of co‐occurring *PIK3CA* and *PTEN* mutations in 6.3% (*n* = 32/508) of the baseline ctDNA samples from SANDPIPER participants (Fig. 1B) may indicate these alterations are derived from different cancer cells in a single tumour or from entirely different lesions. Recently, *FGFR1* alterations have been shown to be associated with worse prognosis compared to *FGFR1* NMD in patients with *PIK3CA*mut tumours treated with ET [35]. Of note, in the same analysis, patients with *PIK3CA*mut tumours that harbour a co‐occurring *FGFR1* alteration experienced improved PFS upon treatment with alpelisib + FUL compared to PBO + FUL [35]. Whereas this latter observation is the opposite association compared to our study, we postulate it may be due in part to the small sample sizes of the biomarker‐positive cohorts, in addition to differences in the timing of sample collection (i.e. freshly collected blood sample vs. archival or freshly collected tumour tissue sample) and methodologies (e.g. blood‐ vs. tissue‐based NGS assays, challenges associated with calling somatic copy number variation in samples with low fractions of ctDNA) used to determine alteration statuses of the participants. Future studies are warranted to further determine the role of *FGFR1* alterations and response to p110α inhibition in *PIK3CA*mut tumours.

Our search for alterations that may be predictive of response to study treatment identified that *PIK3CA*mut participants with a co‐occurring *NF1* alteration in baseline ctDNA experienced significantly improved PFS upon treatment with TAS + FUL compared to PBO + FUL (mPFS: 5.7 vs. 1.9 months; HR = 0.28, 95% CI 0.11–0.67; *q* = 0.017; Figs 2C and 3D). The trend of participants with *NF1* alterations exhibiting shorter PFS compared to participants with *NF1* NMD upon PBO + FUL treatment (mPFS: 1.9 vs. 3.7 months; HR = 2.1, 95% CI 1.1–4.1; *P* = 0.053; *q* = 0.35; Fig. 3D, Fig. S3A) is consistent with prior studies that have identified neurofibromin (NF1) inactivation as a resistance mechanism to ET [3, 27, 36]. Preclinical data have shown that NF1 functions as a dual repressor of Ras signalling and ER transcriptional activity, and the loss of *NF1* results in oestradiol hypersensitivity, contributing to ET resistance [37]. Our analysis suggests that PI3K inhibition may overcome the negative impact of *NF1* loss in ER+, HER2− advanced breast cancers. While the mechanism remains to be elucidated in breast cancer, prior work suggests that NF1 regulates the proliferation of neural stem cells in a PI3K‐dependent manner [38].

To expand beyond genomic alterations, we evaluated the association between ctDNA levels and clinical outcome. We found that *PIK3CA* mut participants tended to have higher baseline TFE compared to *PIK3CA* NMD participants. In addition, we demonstrated that participants with high baseline TFE experienced worse PFS compared to participants with low baseline TFE across study treatments, regardless of *PIK3CA* mutation status in baseline ctDNA. This association between high baseline ctDNA levels and worse clinical outcomes was similarly observed in patients in the PALOMA‐3 study (NCT01942135) [17], EVE biomarker study (NCT02109913) [39], and in samples collected from an independent cohort of patients with ER+, HER2− metastatic breast cancer [40]. Moreover, this association has been demonstrated in patients with breast cancer in the neoadjuvant [41] and adjuvant settings [42], as well as across multiple cancer types [43]. The prognostic value of ctDNA is thought to be partly the result of being detectable upon tumour cell shedding into the bloodstream [11]; as such, the quantity of ctDNA may be indicative of tumour burden and/or aggressiveness [7, 43, 44, 45].

Lastly, we evaluated the scope of newly detected alterations in EOT samples from participants who exhibited clinical benefit in order to identify biomarkers that may be associated with acquired resistance to study treatment. We focused on the TAS + FUL‐treated *PIK3CA*mut participants who exhibited clinical benefit and clustered genes involved in similar signalling pathways. Newly detected alterations at EOT were predominantly observed in genes associated with the ER, PI3K and p53 signalling pathways (Fig. 6A–C and Fig. S7C), suggesting that the activation or dysregulation of these signalling pathways contributes to clinical resistance to PI3K inhibition plus ET in ER+, HER2− breast tumours. As previously reported, the acquisition of *ESR1* or *TP53* alterations likely reflects resistance to ET [17, 21, 22, 27, 28, 29]. The acquisition of *PTEN* or *AKT1* mutations is likely reflective of resistance to PI3K inhibition [32, 33, 46, 47, 48]. Collectively, these findings underscore the value of longitudinal ctDNA analysis to elucidate changes in PI3K and associated pathways following treatment with pathway‐specific inhibitors.

A key strength of this study is that it leverages one of the largest clinico‐genomic datasets to date of participants with *PIK3CA*mut ER+, HER2− breast cancer treated with a PI3K inhibitor, allowing for detailed subgroup analyses based on genomic alteration status. Nevertheless, our study has several limitations. First, the EOT ctDNA data were derived from the analysis of plasma samples only from *PIK3CA*mut participants who exhibited clinical benefit [11.7% (*n* = 12/103) of PBO + FUL‐treated and 17.8% (*n* = 42/236) of TAS + FUL‐treated]; thus, the resulting interpretation of the EOT landscape may not be reflective of the total randomized population. Second, taselisib treatment is associated with increased toxicities, as illustrated by the higher proportion of participants treated with TAS + FUL who experienced adverse events leading to discontinuation [16.8% (*n* = 70/416) with TAS + FUL vs. 2.3% (*n* = 5/213) with PBO + FUL] [6]. As a result, some participants may have discontinued study treatment for reasons other than disease progression; thus, the association of the evaluated oncogenic drivers with clinical outcomes may be underestimated. Moreover, the sample sizes of some biomarker‐positive subgroups were small, and therefore, the analysis may be underpowered. Lastly, as CDK4/6 inhibitors in combination with ET are approved for use in first‐line HR+, HER2− advanced breast cancer, some may question the applicability of our findings for a CDK4/6 inhibitor‐treated patient population. That said, patients with *PIK3CA*mut HR+, HER2− advanced breast cancer have been shown to benefit from treatment with the p110α inhibitor alpelisib plus fulvestrant, regardless of prior treatment with a CDK4/6 inhibitor [49, 50]. Furthermore, less than 50% of patients with HR+, HER2− advanced breast cancer are prescribed CDK4/6 inhibitors as first‐line treatment [51, 52, 53, 54] despite approvals in the first‐line setting, illustrating the continued relevance of the SANDPIPER population to a real‐world setting.

## 5 Conclusion

Our comprehensive baseline ctDNA analysis characterized the genomic heterogeneity of a previously treated ER+, HER2− advanced breast cancer patient population. Although there are no approved treatment options to directly target the alterations we have identified, the detection of a prognostic biomarker from the cumulative molecular landscape of patients may identify those with high‐risk diseases and who perhaps warrant additional intervention. Moreover, our analysis of paired baseline and EOT ctDNA samples identified potential mechanisms of resistance to the study treatment that is largely driven by alterations in genes associated with the ER, PI3K, p53, and/or FGFR signalling pathways. This has important clinical implications following the use of PI3K inhibitors in the treatment of patients with ER+, HER2− advanced breast cancer.

## Conflict of interest

JWC, TJS, FS, HMS, KEH and TRW are full‐time employees of Genentech/Roche and hold stock in Roche. WJ: Grants, personal fees and non‐financial support from AstraZeneca; personal fees and non‐financial support from Eisai, Novartis, Roche, Pfizer, Eli Lilly and Chugai; personal fees from Merck Sharp & Dohme, Bristol Myers Squibb and Seagen and grants and personal fees from Daiichi Sankyo, outside the submitted work. JC: Consulting/Advisor: Roche, Celgene, Cellestia, AstraZeneca, Seattle Genetics, Daiichi Sankyo, Erytech, Athenex, Polyphor, Lilly, Merck Sharp & Dohme, GSK, Leuko, Bioasis, Clovis Oncology, Boehringer Ingelheim, Ellipses, Hibercell, BioInvent, Gemoab, Gilead, Menarini, Zymeworks, Reveal Genomics; Honoraria: Roche, Novartis, Celgene, Eisai, Pfizer, Samsung Bioepis, Lilly, Merck Sharp & Dohme, Daiichi Sankyo; Research funding to the Institution: Roche, Ariad pharmaceuticals, AstraZeneca, Baxalta GMBH/Servier Affaires, Bayer healthcare, Eisai, F. Hoffman‐La Roche, Guardant Health, Merck Sharp & Dohme, Pfizer, Piqur Therapeutics, Puma C, Queen Mary University of London; Stock: MedSIR, Nektar Pharmaceuticals, Leuko (relative); Travel, accommodation, expenses: Roche, Novartis, Eisai, Pfizer, Daiichi Sankyo, Astrazeneca, Gilead. Patents: ‘Pharmaceutical Combinations of A Pi3k Inhibitor And A Microtubule Destabilizing Agent. Javier Cortés Castán, Alejandro Piris Giménez, Violeta Serra Elizalde. WO 2014/199294 A’ & ‘Her2 as a predictor of response to dual HER2 blockade in the absence of cytotoxic therapy. Aleix Prat, Antonio Llombart, Javier Cortés. US 2019/0338368 A1’. IEK: Honoraria from Genentech, AstraZeneca and Celltrion; fees from a consulting or advisory role from Genentech/Roche, Seattle Genetics, Daiichi Sankyo, MacroGenics, Taiho Pharmaceutical, Context Therapeutics, Novartis, Merck and Ionis; research funding from Genentech and Pfizer; employment/leadership/stock and other ownership interests (for an immediate family member) from AMAG Pharmaceuticals. SD: Honoraria for consulting: Novartis, AstraZeneca. NH: Honoraria for lectures and/or consulting: Amgen, AstraZeneca, Daiichi‐Sankyo, Exact Sciences, Gilead, Lilly, Merck Sharp & Dohme, Novartis, Pierre‐Fabre, Pfizer, Roche, Sandoz, Seagen. MDL: Honoraria for lectures and/or consulting from: Astrazeneca, Amgen, Celgene, Daiichi Sankyo, Eisai, Eli Lilly, Exact Science, Gilead, Merck Sharp & Dohme, Novartis, Pfizer, Pierre Fabre, Roche, Seagen. VD: Honoraria for lectures and/or consulting: Roche/Genentech, Novartis, Lilly, Pfizer, AstraZeneca, AbbVie, Merck Sharp & Dohme, Daiichi Sankyo, SEAGEN, Gilead, Eisai, Pierre Fabre Oncologie. YHI: No conflicts of interest.

## Author contributions

KEH, TRW and JWC were involved in conception and design. WJ, JC, IEK, SD, NH, MDL, VD, Y‐HI, HMS and TRW carried out acquisition of data. HMS, JWC, KEH, TRW, TJS and FS carried out analysis and interpretation of data. JWC, WJ, JC, IEK, SD, NH, MDL, VD, Y‐HI, TJS, FS, HMS, KEH and TRW carried out writing, review, and/or revision of the manuscript. HMS and TRW were involved in administrative, technical or material support. KEH and TRW carried out study supervision.

### Peer review

The peer review history for this article is available at https://www.webofscience.com/api/gateway/wos/peer‐review/10.1002/1878‐0261.13416.

## Supporting information


**Fig. S1.** Frequency of *PIK3CA* single‐nucleotide variants (SNVs) detected in baseline ctDNA.

**Fig. S2.** Genomic landscape of baseline ctDNA from participants with (A, B) *PIK3CA*mut and (C–E) *PIK3CA* NMD, ER+, HER2− advanced breast cancer.

**Fig. S3.** Association of progression‐free survival (PFS) with genomic alteration status in participants with (A) *PIK3CA*mut baseline ctDNA and (B) *PIK3CA* NMD baseline ctDNA.

**Fig. S4.** Association of PFS with (A, B) genomic alteration status and (C) study treatment in participants with *PIK3CA* NMD baseline ctDNA.

**Fig. S5.** Kaplan–Meier plots of PFS per treatment arm and alteration status for *PIK3CA* NMD cohort.

**Fig. S6** Association between TFE and PFS in participants with *PIK3CA* NMD baseline ctDNA.

**Fig. S7.** Genetic landscape of breast cancer tumours at EOT in participants with *PIK3CA*mut baseline ctDNA who exhibited clinical benefit.

**Fig. S8.** Genetic landscape of breast cancer tumours at EOT in participants with *PIK3CA* NMD baseline ctDNA who exhibited clinical benefit.

**Table S1.** Participant demographics and disease characteristics stratified by *PIK3CA*mut status per baseline ctDNA and by treatment arm.

**Table S2.** Summary of *PIK3CA* mutations (*PIK3CA*mut) identified in baseline ctDNA per the F1L assay beyond the mutation coverage of the cobas^®^ *PIK3CA* Mutation Test.

**Data S1.** Legends.

## Data Availability

All relevant data are provided in the main text or the supplementary materials. Information on the availability of data from the SANDPIPER clinical trial can be found in the published primary clinical manuscript [6]. For up‐to‐date details on Roche's Global Policy on the Sharing of Clinical Information and how to request access to related clinical study documents, see here: https://go.roche.com/data_sharing.
