# Supplementary figures and images for: ER+, HER2− advanced breast cancer treated with taselisib and fulvestrant: genomic landscape and associated clinical outcomes
Source: Mol Oncol. 2023 Mar 25;17(10):2000–16. doi: 10.1002/1878-0261.13416 (PMC10552898; doi:10.1002/1878-0261.13416)

SUPPLEMENTARY FIGURE S1

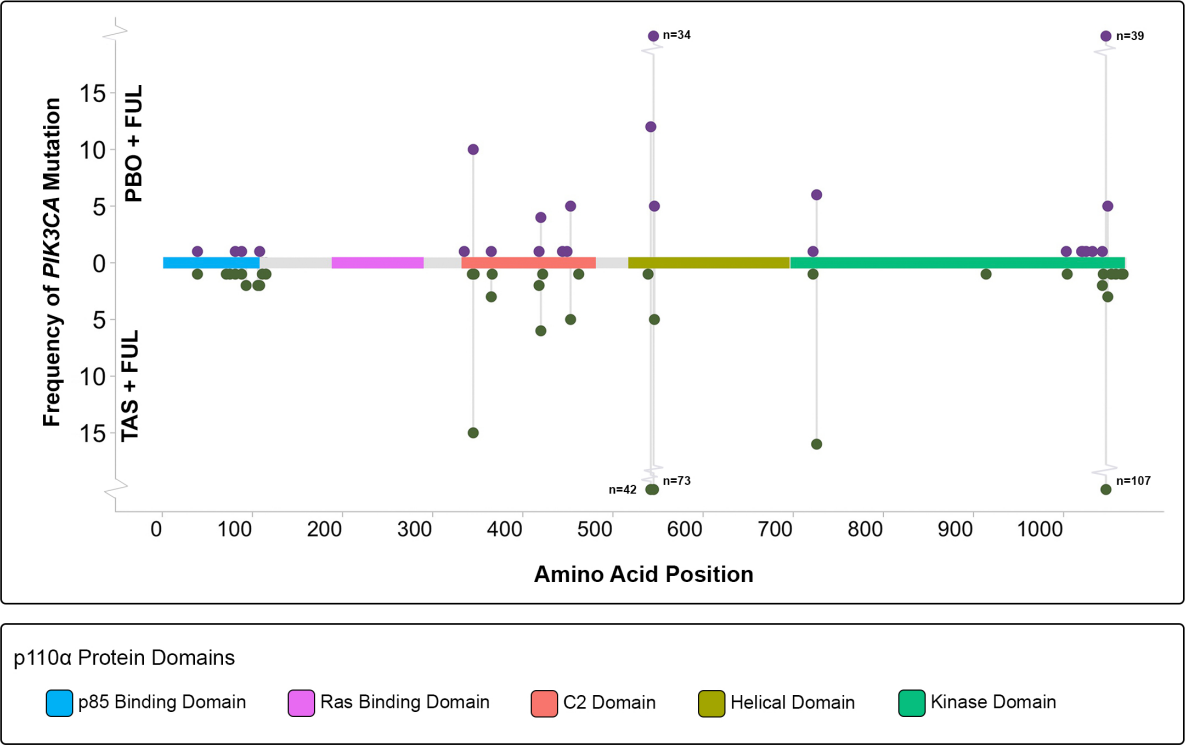

Supplement: Supplementary file 1 — Fig. S1. Frequency of PIK3CA single‐nucleotide variants (SNVs) detected in baseline ctDNA. [file MOL2-17-2000-s005.pdf]

FIGURE S2

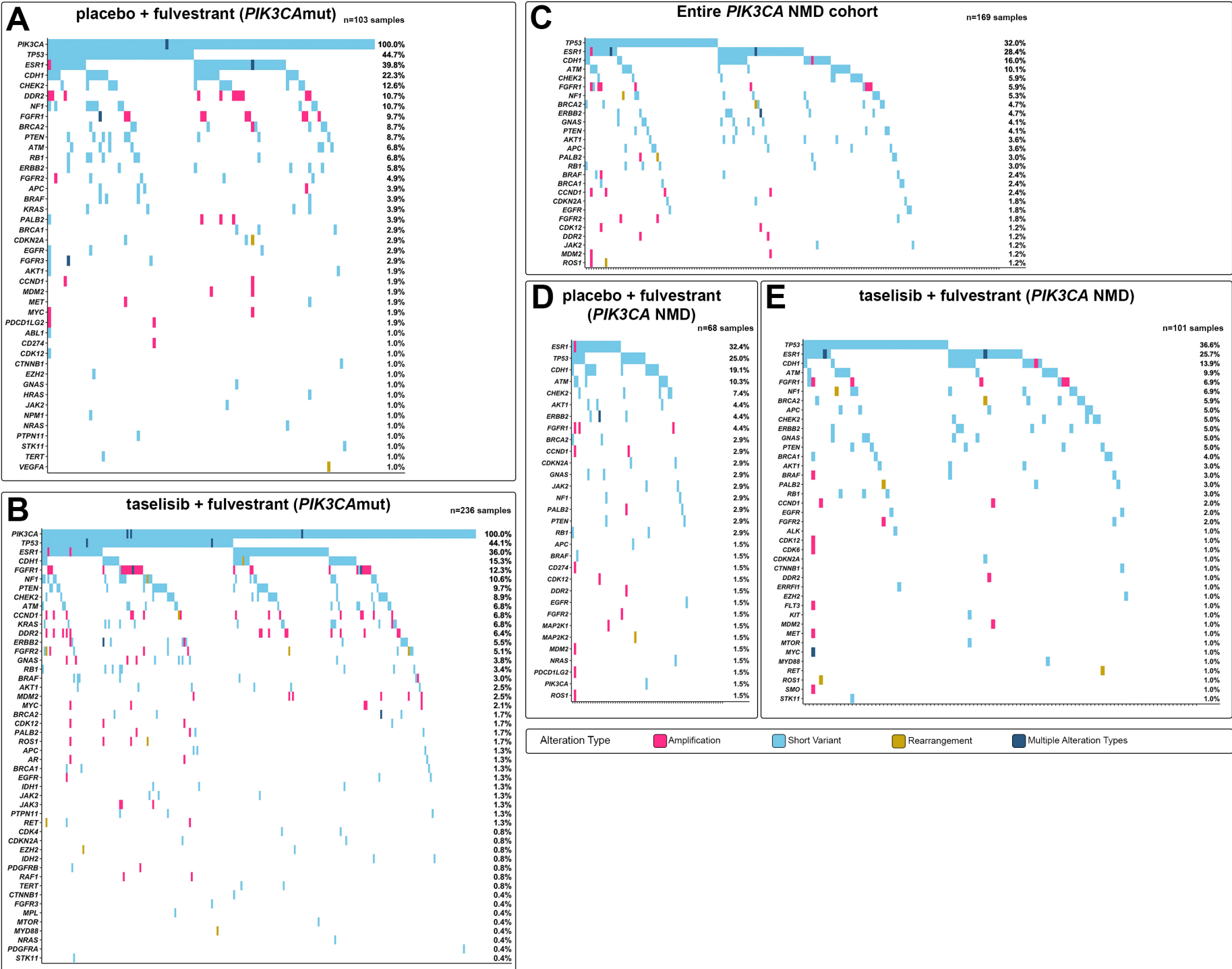

Supplement: Supplementary file 2 — Fig. S2. Genomic landscape of baseline ctDNA from participants with (A, B) PIK3CAmut and (C–E) PIK3CA NMD, ER+, HER2− advanced breast cancer. [file MOL2-17-2000-s003.pdf]

FIGURE S3

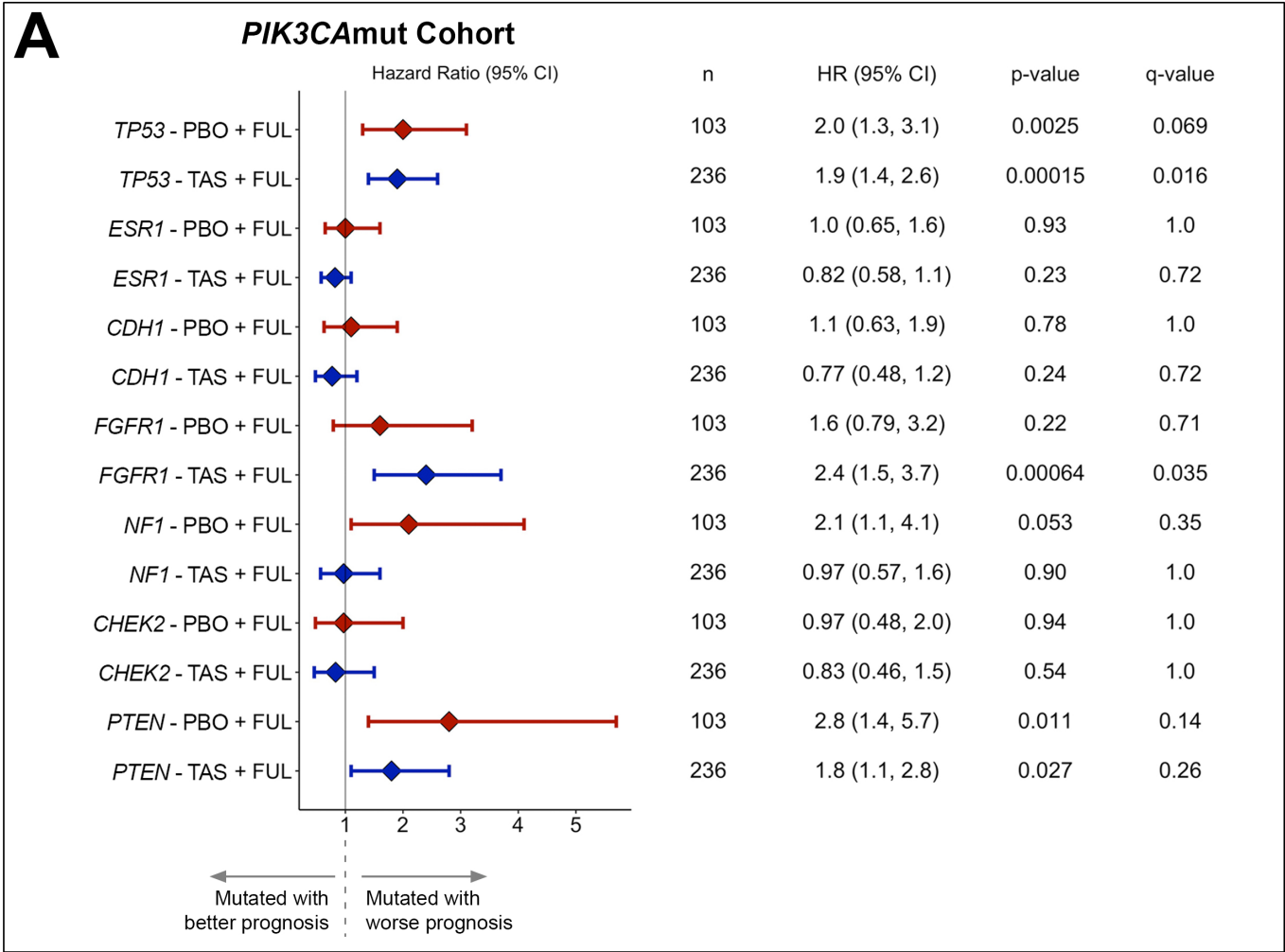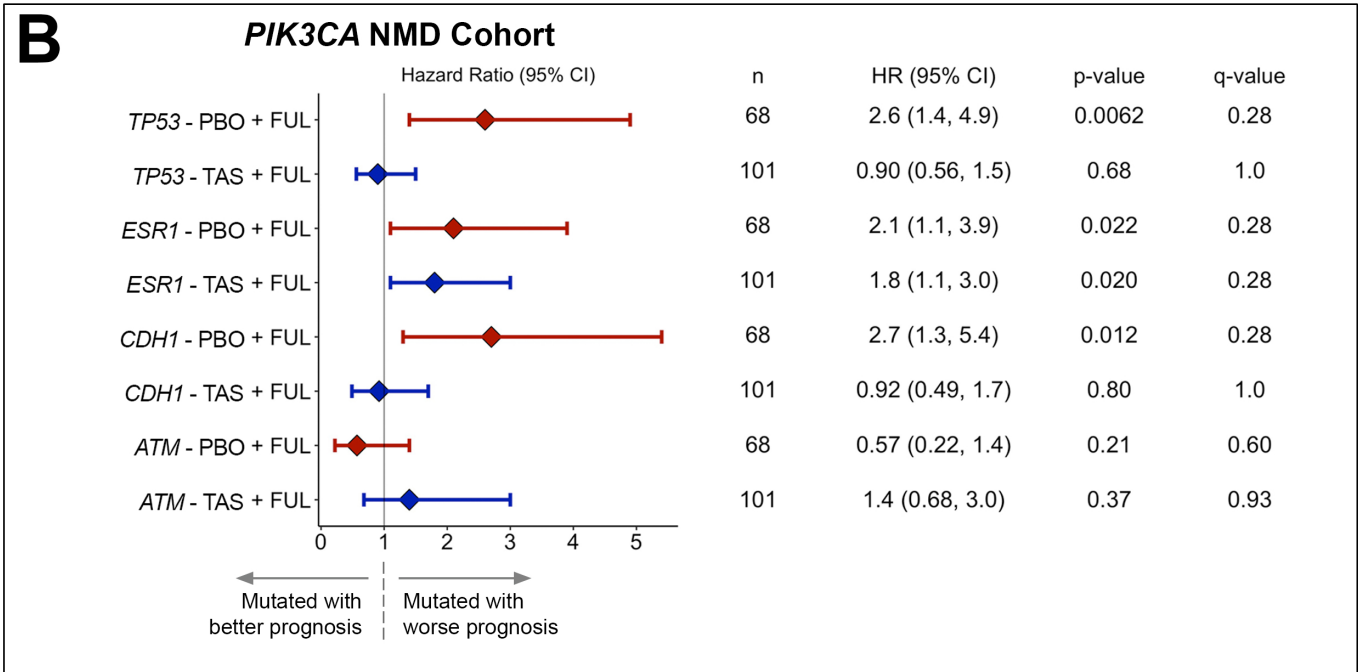

Supplement: Supplementary file 3 — Fig. S3. Association of progression‐free survival (PFS) with genomic alteration status in participants with (A) PIK3CAmut baseline ctDNA and (B) PIK3CA NMD baseline ctDNA. [file MOL2-17-2000-s006.pdf]

FIGURE S4

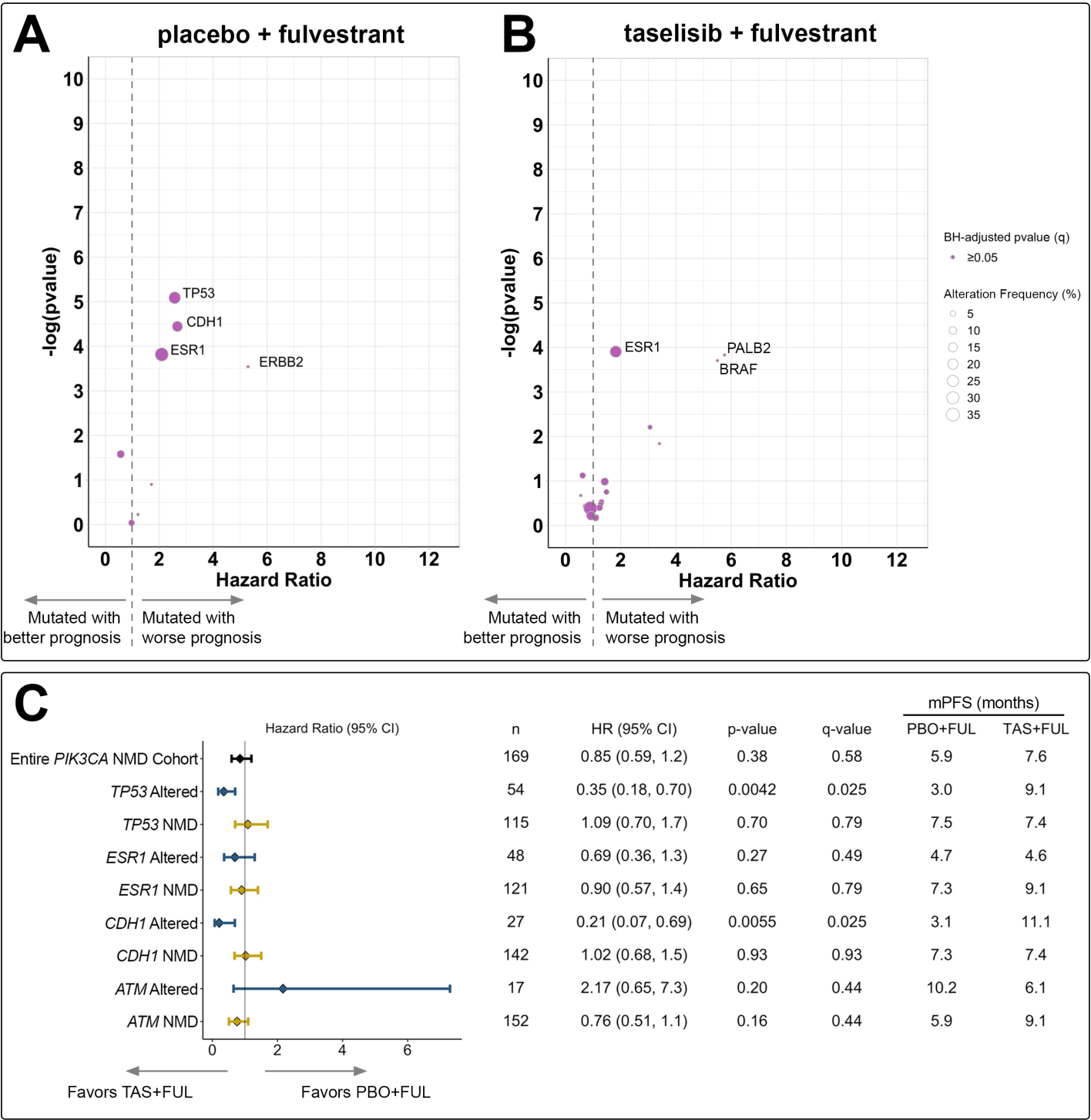

Supplement: Supplementary file 4 — Fig. S4. Association of PFS with (A, B) genomic alteration status and (C) study treatment in participants with PIK3CA NMD baseline ctDNA. [file MOL2-17-2000-s009.pdf]

FIGURE S5

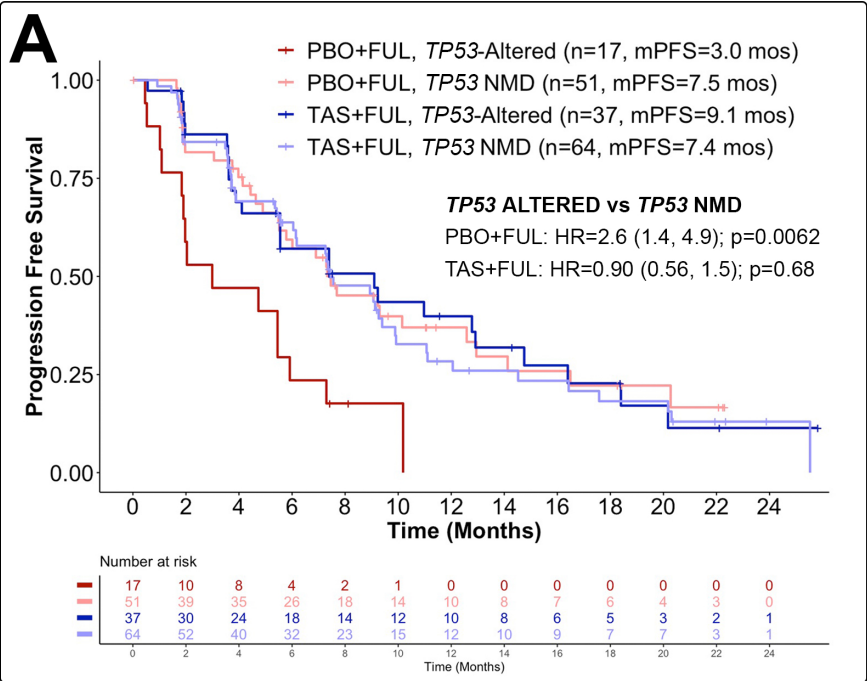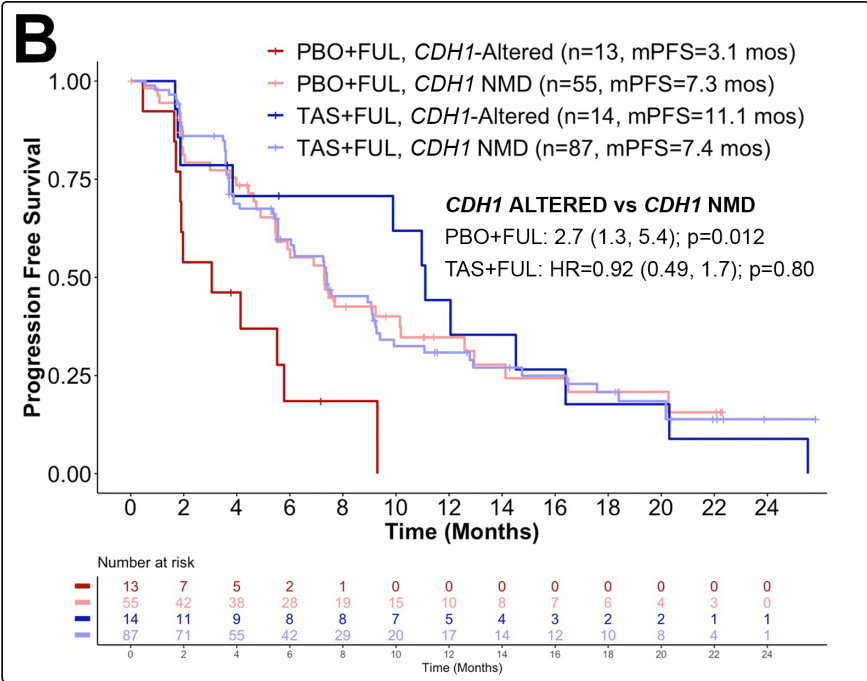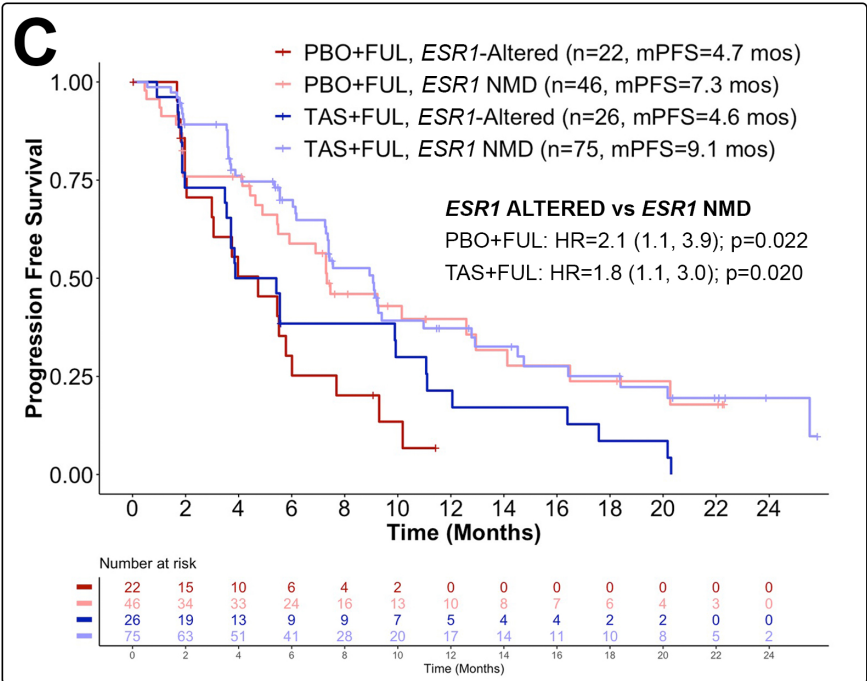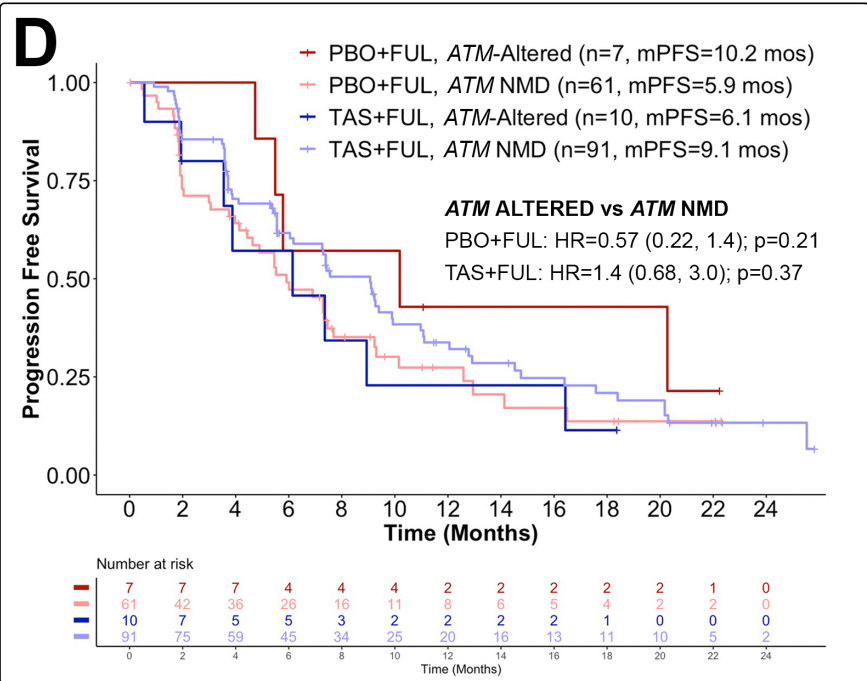

Supplement: Supplementary file 5 — Fig. S5. Kaplan–Meier plots of PFS per treatment arm and alteration status for PIK3CA NMD cohort. [file MOL2-17-2000-s010.pdf]

FIGURE S6

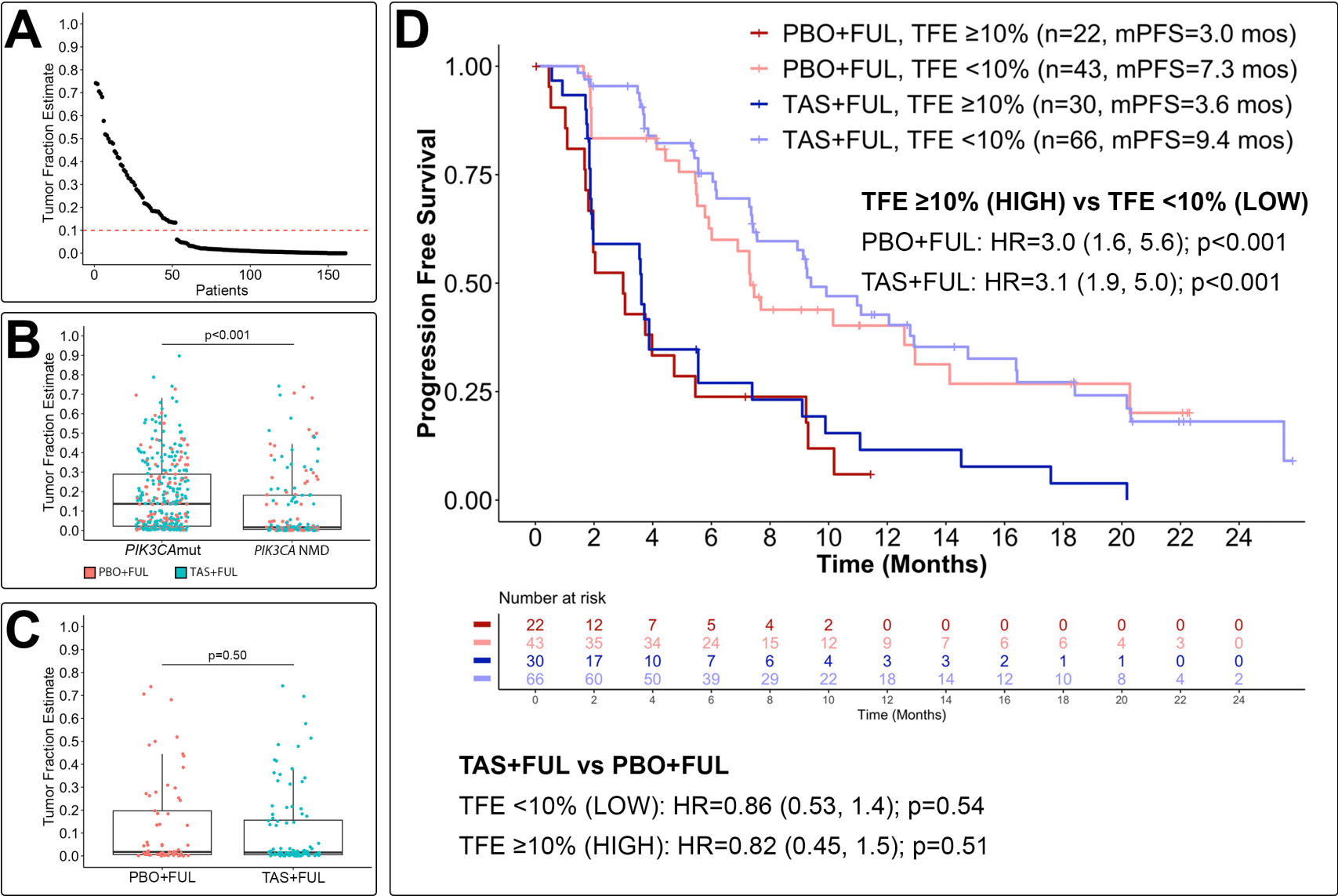

Supplement: Supplementary file 6 — Fig. S6 Association between TFE and PFS in participants with PIK3CA NMD baseline ctDNA. [file MOL2-17-2000-s001.pdf]

**FIGURE S7**

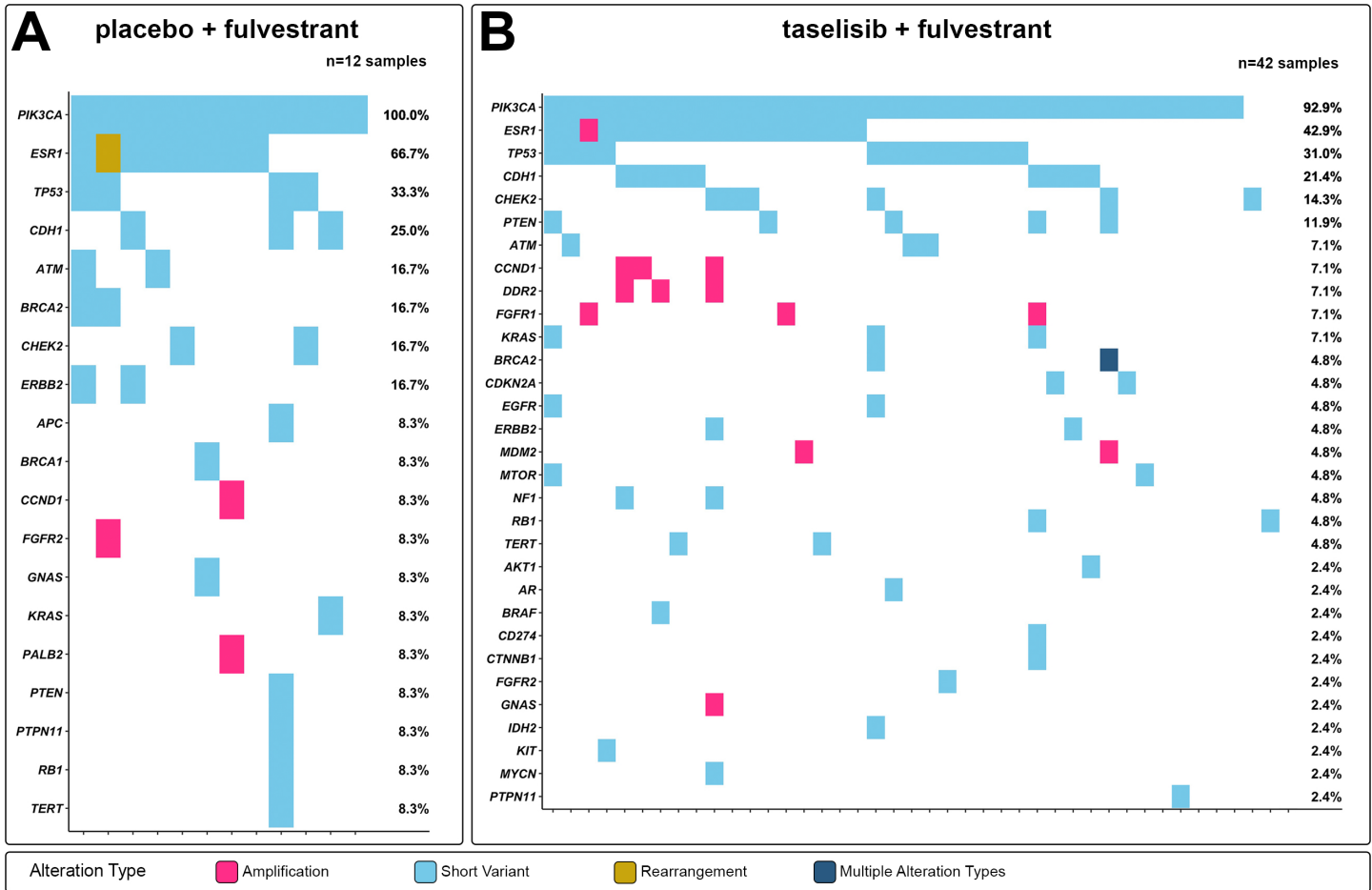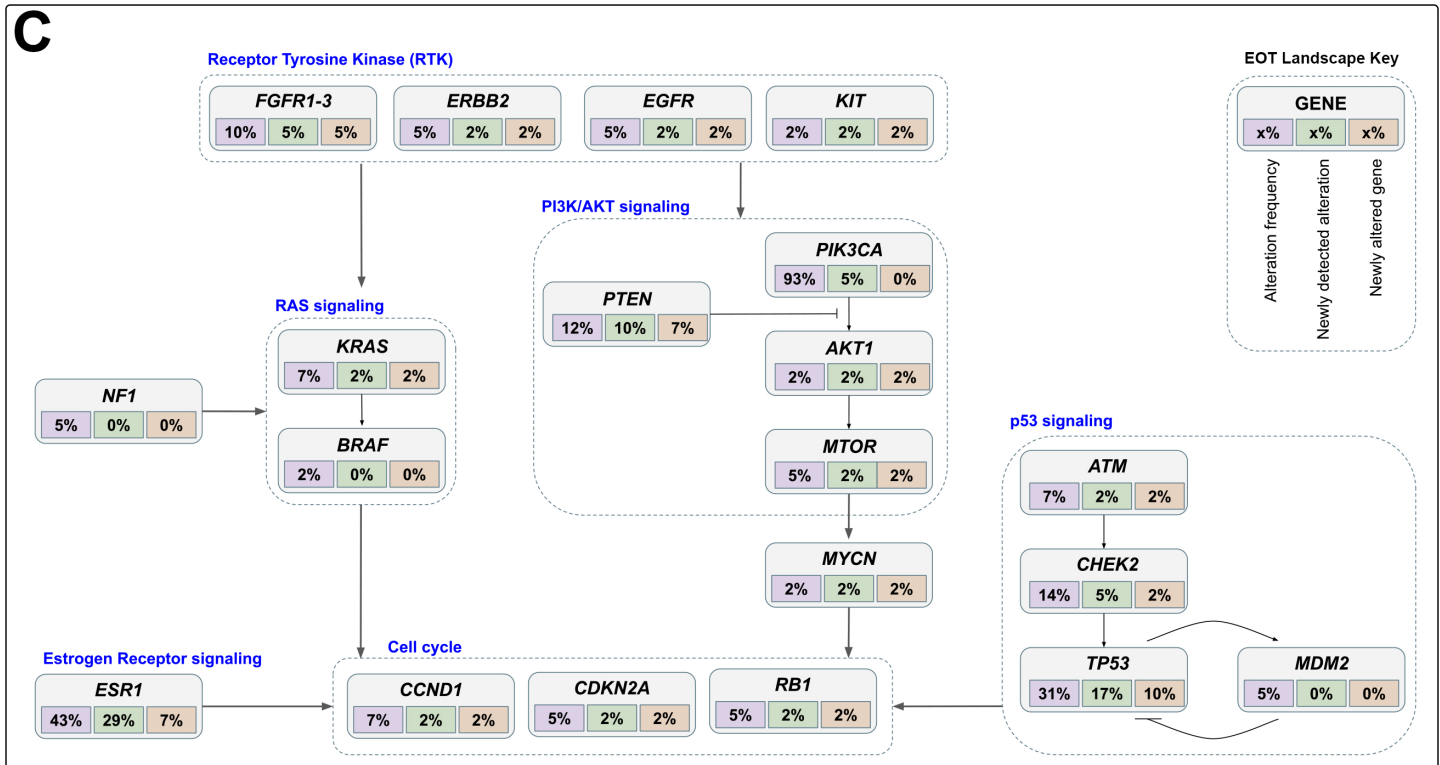

Supplement: Supplementary file 7 — Fig. S7. Genetic landscape of breast cancer tumours at EOT in participants with PIK3CAmut baseline ctDNA who exhibited clinical benefit. [file MOL2-17-2000-s011.pdf]

FIGURE S8

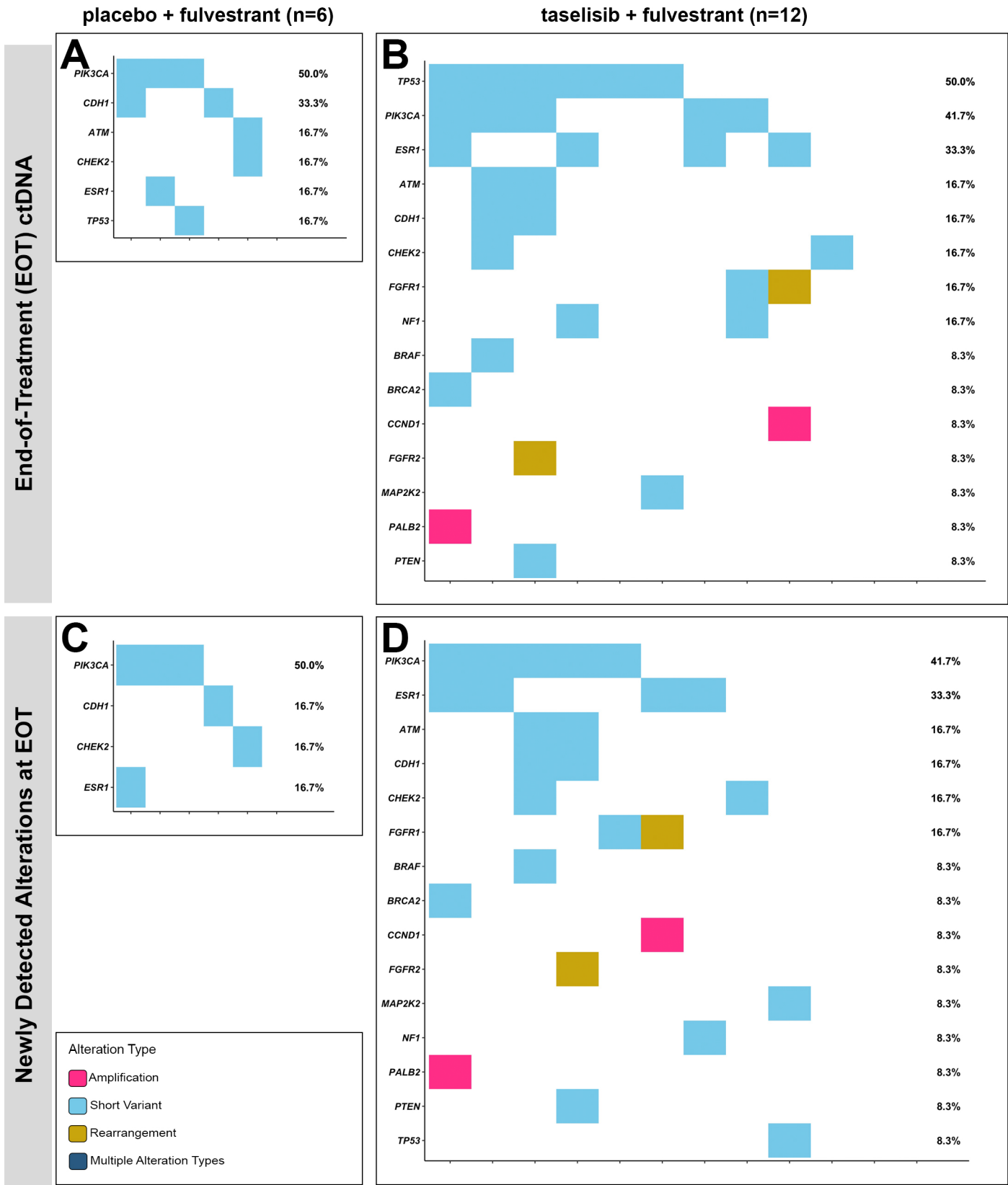

Supplement: Supplementary file 8 — Fig. S8. Genetic landscape of breast cancer tumours at EOT in participants with PIK3CA NMD baseline ctDNA who exhibited clinical benefit. [file MOL2-17-2000-s007.pdf]
