# Supplementary material for: ER+, HER2− advanced breast cancer treated with taselisib and fulvestrant: genomic landscape and associated clinical outcomes
Source: Mol Oncol. 2023 Mar 25;17(10):2000–16. doi: 10.1002/1878-0261.13416 (PMC10552898; doi:10.1002/1878-0261.13416)
Supplement: Supplementary file 9 — Table S1. Participant demographics and disease characteristics stratified by PIK3CAmut status per baseline ctDNA and by treatment arm. [file MOL2-17-2000-s004.pdf]

TABLE S1. Participant demographics and disease characteristics stratified by *PIK3CA*mut status per baseline ctDNA and by treatment arm.

	*PIK3CA*mut (n=339)		*PIK3CA* NMD (n=169)		p-value[a]	BH-adjusted p-value (q-value)
	PBO+FUL n=103	TAS+FUL n=236	PBO+FUL n=68	TAS+FUL n=101		
Age in years, median (range)	60 (34 - 85)	60 (32 - 84)	62 (39 - 85)	61 (39 - 83)	0.53[b]	0.81
ECOG PS (n, %)						
0	52 (50.5%)	125 (53.0%)	38 (55.9%)	62 (61.4%)	0.41	0.81
1	51 (49.5%)	111 (47.0%)	30 (44.1%)	39 (38.6%)		
Visceral disease (n, %)	61 (59.2%)	148 (62.7%)	41 (60.3%)	64 (63.4%)	0.91	0.91
Bone-only disease	15 (14.6%)	45 (19.1%)	17 (25.0%)	20 (19.8%)	0.40	0.81
Bone metastasis	77 (74.8%)	194 (82.2%)	50 (73.5%)	69 (68.3%)	0.033	0.56
Measurable disease (n, %)	82 (79.6%)	188 (79.7%)	49 (72.1%)	76 (75.2%)	0.50	0.81
Endocrine sensitivity	68 (66.0%)	170 (72.0%)	54 (79.4%)	70 (69.3%)	0.28	0.81
Prior endocrine therapy						
Prior adjuvant ET	68 (66.0%)	145 (61.4%)	42 (61.8%)	62 (61.4%)	0.87	0.917
Prior ET for mBC	67 (65.0%)	176 (74.6%)	54 (79.4%)	71 (70.3%)	0.16	0.81
Prior tamoxifen (regardless of setting)	44 (42.7%)	116 (49.2%)	38 (55.9%)	49 (48.5%)	0.41	0.81
Prior CDK4/6 inhibitor	3 (2.9%)	9 (3.8%)	1 (1.5%)	4 (4.0%)	0.87	0.91
Prior chemotherapy						
Prior chemotherapy in mBC	31 (30.1%)	75 (31.8%)	23 (33.8%)	27 (26.7%)	0.75	0.91

Prior systemic therapy in mBC	74 (71.8%)	185 (78.4%)	54 (79.4%)	74 (73.3%)	0.33	0.81
Number of regimens in mBC, median (IQR)<br>Range	1 (0 - 2)<br>0 - 4	1 (1 - 2)<br>0 - 5	1 (1 - 2)<br>0 - 6	1 (0 - 2)<br>0 - 7	0.34[b]	0.81
Region						
Western Europe/ USA/ Australia	59 (57.3%)	119 (50.4%)	35 (51.4%)	56 (55.4%)	0.64	0.91
Asia	8 (7.8%)	32 (13.6%)	8 (11.8%)	14 (13.9%)	0.45	0.81
Rest of the world	36 (34.9%)	85 (36.0%)	25 (36.8%)	31 (30.7%)	0.80	0.91

[a]p-value is based on two-sided Fisher's Exact Test unless denoted otherwise. [b]p-value based on Kruskal-Wallis Test. CDK, cyclin-dependent kinase; ECOG PS, Eastern Cooperative Oncology Group Performance Status; ET, endocrine therapy; IQR, interquartile range; mBC, metastatic breast cancer; n, sample size.
