# Supplementary material for: ER+, HER2− advanced breast cancer treated with taselisib and fulvestrant: genomic landscape and associated clinical outcomes
Source: Mol Oncol. 2023 Mar 25;17(10):2000–16. doi: 10.1002/1878-0261.13416 (PMC10552898; doi:10.1002/1878-0261.13416)
Supplement: Supplementary file 10 — Table S2. Summary of PIK3CA mutations (PIK3CAmut) identified in baseline ctDNA per the F1L assay beyond the mutation coverage of the cobas® PIK3CA Mutation Test. [file MOL2-17-2000-s008.pdf]

TABLE S2. Summary of *PIK3CA* mutations identified in baseline ctDNA per the F1L assay beyond the mutation coverage of the cobas® *PIK3CA* Mutation Test. Both pathogenic variants (i.e. predicted to be of known or likely oncogenic significance) and variants of unknown significance are listed.

***PIK3CA* mutation**	**# Baseline Samples**
E726K	22
E453K	6
E365K	4
E453Q	4
E418K	3
E542Q	3
R108H	3
E39K	2
E81K	2
E722K	2
E542A	1
E545Q	1
G1049S	1
G106V	1
I1058L	1
K111N	1
M1004I	1
N1044S	1
P539R	1
Q546H	1
Q546P	1
Q75E	1
R115L	1
R93Q	1
R93W	1
T1025A	1
Y1021H	1
A1020E	1

A1066I	1
E1032K	1
G106D	1
G914R	1
L422W	1
N444K	1
P366R	1
P449S	1
Q1064H	1
R335T	1
S1003L	1
T1053A	1
T462A	1
V344E	1
V346E	1
V71I	1
