# Supplementary material for: ER+, HER2− advanced breast cancer treated with taselisib and fulvestrant: genomic landscape and associated clinical outcomes
Source: Mol Oncol. 2023 Mar 25;17(10):2000–16. doi: 10.1002/1878-0261.13416 (PMC10552898; doi:10.1002/1878-0261.13416)
Supplement: Supplementary file 11 — Data S1. Legends. [file MOL2-17-2000-s002.docx]

**SUPPLEMENTAL FIGURE LEGENDS**

**FIGURE S1. Frequency of *PIK3CA* single nucleotide variants (SNVs) detected in baseline ctDNA.** *PIK3CA*mut is defined by the presence of a detectable pathogenic (i.e., predicted to be of known or likely oncogenic significance) *PIK3CA* SNV in baseline ctDNA. *PIK3CA* SNVs were detected in 60.8% (n=104/171) PBO+FUL-treated and 70.3% (n=237/337) TAS+FUL-treated participants. Hotspot mutations were present at amino acids E542, E545, and H1047. Both pathogenic variants and variants of unknown significance are shown in the dumbbell plot. ctDNA, circulating tumor DNA: FUL, fulvestrant; mut, mutated; n, sample size; p110α, phosphatidylinositol-4,5-bisphosphate 3-kinase catalytic subunit alpha; PBO, placebo; TAS, taselisib.

**FIGURE S2. Genomic landscape of baseline ctDNA from participants with (A-B) *PIK3CA*mut and (C-E) *PIK3CA* NMD, ER+, HER2– advanced breast cancer.** Genomic landscape of baseline ctDNA from (A) PBO+FUL-treated *PIK3CA*mut participants, (B) TAS+FUL-treated *PIK3CA*mut participants, (C) the entire *PIK3CA* NMD cohort, (D) PBO+FUL-treated *PIK3CA* NMD participants, and (D) TAS+FUL-treated *PIK3CA* NMD participants. Individual samples may harbor ≥1 alteration of the same variant type in a single gene (e.g., ≥1 *TP53* short variants); this information is not denoted in the tile plots. ctDNA, circulating tumor DNA; ER, estrogen receptor; FUL, fulvestrant; HER2, Human epidermal growth factor receptor 2; mut, mutated; n, sample size; NMD, no mutation detected; PBO, placebo; TAS, taselisib.

**FIGURE S3. Association of progression-free survival (PFS) with genomic alteration status in participants with (A) *PIK3CA*mut baseline ctDNA and (B) *PIK3CA* NMD baseline ctDNA.** Only the top altered genes (altered in ≥10% of samples) in each respective cohort are shown in the forest plots. Log-rank tests using a Cox proportional hazards regression model were used to obtain hazard ratio (HR) and p-values; statistical significance is defined by Benjamini-Hochberg adjusted p-value (q-value) <0.05. CI, confidence interval; ctDNA, circulating tumor DNA; FUL, fulvestrant; mut, mutated; n, sample size; NMD, no mutation detected; PBO, placebo; TAS, taselisib.

**FIGURE S4. Association of PFS with (A-B) genomic alteration status and (C) study treatment in participants with *PIK3CA* NMD baseline ctDNA.** Association between progression-free survival (PFS) and genomic alteration status in (A) PBO+FUL-treated participants and (B) TAS+FUL-treated participants. Only genes that were altered in ≥1% of samples (n≥3) are shown in the volcano plots; the gene is annotated if the nominal p-value <0.05. The size of the bubble indicates the frequency of the alterations in the respective gene within the treatment arm. (C) PFS by biomarker status of the top altered genes (altered in ≥10% of samples). Log-rank tests using a Cox proportional hazards regression model were used to obtain hazard ratio (HR) and p-values; statistical significance is defined by Benjamini-Hochberg adjusted p-value (q-value) <0.05. CI, confidence interval; ctDNA, circulating tumor DNA; FUL, fulvestrant; mPFS, median progression-free survival; mut, mutated; n, sample size; NMD, no mutation detected; PBO, placebo; TAS, taselisib.

**FIGURE S5. Kaplan-Meier plots of PFS per treatment arm and alteration status for *PIK3CA* NMD cohort.** Progression-free survival (PFS) by detectable (A) *TP53*, (B) *CDH1*, (C) *ESR1*, and (D) *ATM* alteration(s) in baseline circulating tumor DNA (ctDNA) for *PIK3CA* NMD participants in the PBO+FUL and TAS+FUL arms. Tick marks indicate censoring events. Log-rank tests using a Cox proportional hazards regression model were used to obtain hazard ratio (HR) and p-values. ATM, ataxia-telangiectasia mutated; CDH1, cadherin-1; ESR1, estrogen receptor 1; FUL, fulvestrant; mPFS, median progression-free survival; mos, months; n, sample size; NMD, no mutation detected; PBO, placebo; TAS, taselisib; TP53, tumor protein p53.

**FIGURE S6. Association between TFE and PFS in participants with *PIK3CA* NMD baseline ctDNA.** (A) Distribution of tumor fraction estimate (TFE) at baseline. Red dashed line (TFE=0.1) denotes cut-off for high TFE (≥10%) versus low TFE (<10%). (B) Distribution of TFE at baseline stratified by *PIK3CA* mutation status. Amongst participants with *PIK3CA*mut baseline ctDNA, the median TFE was 0.14 (IQR 0.02-0.29); amongst participants with *PIK3CA* NMD baseline ctDNA, the median TFE was 0.02 (IQR 0.01-0.18). (C) Distribution of TFE at baseline stratified by study treatment. Amongst TAS+FUL-treated participants, the median TFE was 0.01 (IQR 0.01-0.16); amongst PBO+FUL-treated participants, the median TFE was 0.02 (IQR 0.01-0.20). p-values were obtained from a Wilcoxon rank-sum test. (C) Association between TFE at baseline and PFS. Log-rank tests using a Cox proportional hazards regression model were used to obtain hazard ratio (HR) and p-values. ctDNA, circulating tumor DNA; FUL, fulvestrant; IQR, interquartile range; mPFS, median progression-free survival; mos, months; mut, mutated; NMD, no mutation detected; PBO, placebo; PFS, progression-free survival; TAS, taselisib.

**FIGURE S7. Genetic landscape of breast cancer tumors at EOT in participants with *PIK3CA*mut baseline ctDNA who exhibited clinical benefit.** Genomic landscape of end-of-treatment (EOT) ctDNA from participants upon (A) PBO+FUL and (B) TAS+FUL treatment. Individual samples may harbor ≥1 alteration of the same variant type in a single gene (e.g., ≥1 *TP53* short variants); this information is not denoted in the tile plots. (C) Summary of genetic landscape of breast cancer tumors at EOT in TAS+FUL-treated participants (n=42). Violet-colored boxes indicate the percentage of participants with any alterations in the respective gene at EOT. Green-colored boxes indicate the subset of participants with new alteration(s) in the respective gene that were not detected at baseline (but the same gene may have been differently altered at baseline). Orange-colored boxes indicate the subset of participants with alteration(s) in the respective gene when none were detected at baseline. ctDNA, circulating tumor DNA; FUL, fulvestrant; n, sample size; PBO, placebo; TAS, taselisib.

**FIGURE S8. Genetic landscape of breast cancer tumors at EOT in participants with *PIK3CA* NMD baseline ctDNA who exhibited clinical benefit.** Genomic landscape of end-of-treatment (EOT) circulating tumor DNA (ctDNA) from participants upon (A) PBO+FUL and (B) TAS+FUL treatment. Genomic landscape of newly detected alterations at EOT in participants treated with (C) PBO+FUL and (D) TAS+FUL. Newly detected alterations are defined as alterations that were not detected in baseline ctDNA but were detected in EOT ctDNA. Individual samples may harbor ≥1 alteration of the same variant type in a single gene (e.g., ≥1 *TP53* short variants); this information is not denoted in the tile plots. FUL, fulvestrant; n, sample size; NMD, no mutation detected; PBO, placebo; TAS, taselisib.
